# Y-Chromosomal Diversity in Lebanon Is Structured by Recent Historical Events

**DOI:** 10.1016/j.ajhg.2008.01.020

**Published:** 2008-04-04

**Authors:** Pierre A. Zalloua, Yali Xue, Jade Khalife, Nadine Makhoul, Labib Debiane, Daniel E. Platt, Ajay K. Royyuru, Rene J. Herrera, David F. Soria Hernanz, Jason Blue-Smith, R. Spencer Wells, David Comas, Jaume Bertranpetit, Chris Tyler-Smith, The Genographic Consortium

**Affiliations:** 1 The Lebanese American University, Chouran, Beirut 1102 2801, Lebanon; 2 The Wellcome Trust Sanger Institute, Wellcome Trust Genome Campus, Hinxton, Cambs, CB10 1SA, UK; 3 Bioinformatics and Pattern Discovery, IBM T. J. Watson Research Center, Yorktown Hgts, NY 10598, USA; 4 Department of Biological Sciences, Florida International University, Miami, FL 33199, USA; 5 The Genographic Project, National Geographic Society, Washington, DC 20036, USA; 6 Unitat de Biologia Evolutiva, Departament de Ciènces Experimentals i de la Salut, Universitat Pompeu Fabra, Doctor Aiguader 88, 08003 Barcelona, Catalonia, Spain

## Abstract

Lebanon is an eastern Mediterranean country inhabited by approximately four million people with a wide variety of ethnicities and religions, including Muslim, Christian, and Druze. In the present study, 926 Lebanese men were typed with Y-chromosomal SNP and STR markers, and unusually, male genetic variation within Lebanon was found to be more strongly structured by religious affiliation than by geography. We therefore tested the hypothesis that migrations within historical times could have contributed to this situation. Y-haplogroup J^∗^(xJ2) was more frequent in the putative Muslim source region (the Arabian Peninsula) than in Lebanon, and it was also more frequent in Lebanese Muslims than in Lebanese non-Muslims. Conversely, haplogroup R1b was more frequent in the putative Christian source region (western Europe) than in Lebanon and was also more frequent in Lebanese Christians than in Lebanese non-Christians. The most common R1b STR-haplotype in Lebanese Christians was otherwise highly specific for western Europe and was unlikely to have reached its current frequency in Lebanese Christians without admixture. We therefore suggest that the Islamic expansion from the Arabian Peninsula beginning in the seventh century CE introduced lineages typical of this area into those who subsequently became Lebanese Muslims, whereas the Crusader activity in the 11^th^–13^th^ centuries CE introduced western European lineages into Lebanese Christians.

## Introduction

Compared with other ape species, humans show little genetic variation, despite their much larger population size and wider distribution, and this limited variation can mostly be explained by geographical factors.^1^ Human populations, however, can be classified in many other ways, such as by language, ethnicity, or religion. Populations in which these alternative factors have had a greater influence than geography on the distribution of genetic variation are unusual and merit particular attention. Here, we describe the genetic structure of the peoples of Lebanon, show that religion has had a strong influence on current patterns of patrilineal variation, and identify historical events that might underlie this unusual situation.

**Figure 1:**
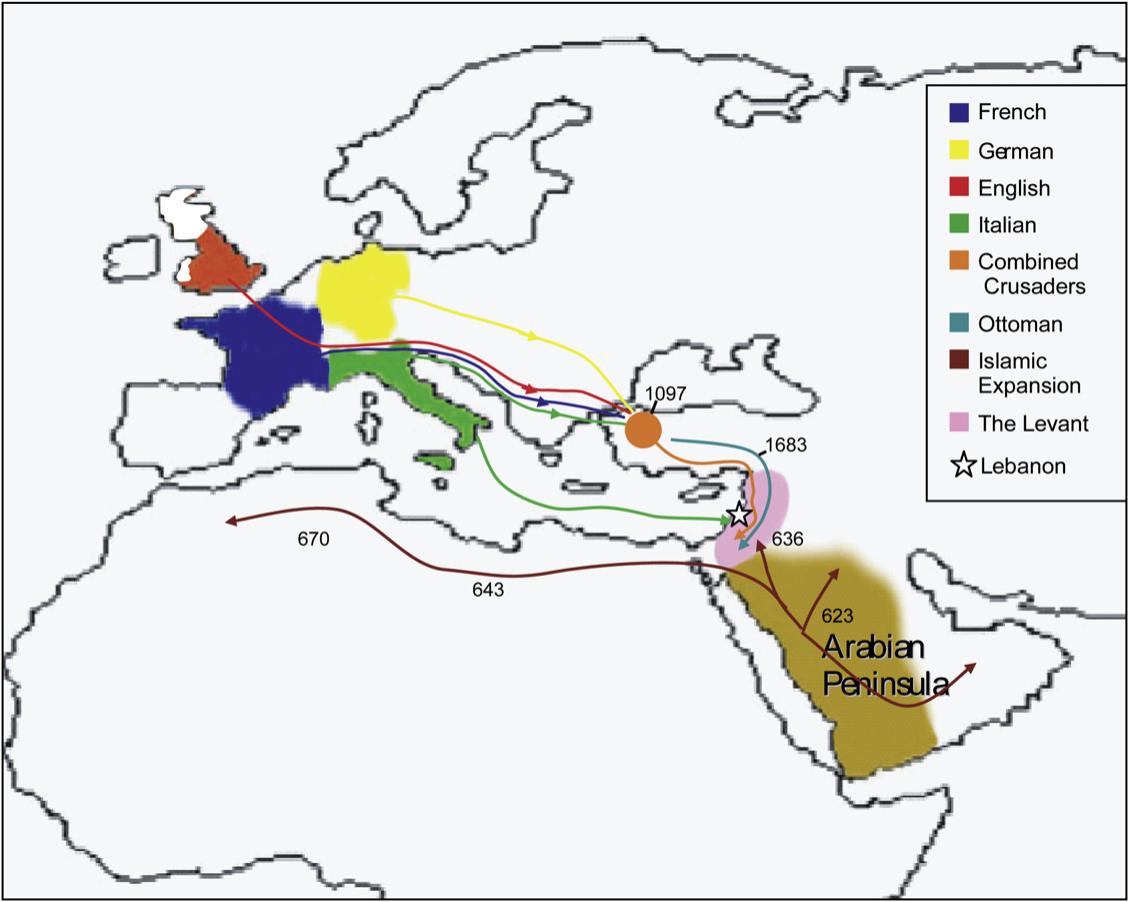
Map of Lebanon and Its Surrounding Regions Showing Historically Documented Migrations into Lebanon

Lebanon is a small country on the eastern coast of the Mediterranean (Figure 1). Just 4,015 square miles in area, it is 1/60th the size of Texas and half the size of Wales. This region was first occupied by fully modern humans ∼47,000 years ago^1^ and appears to have remained habitable even during the unfavorable conditions of the last glacial maximum 18,000–21,000 years ago.^2^ It is close to the Fertile Crescent where the West Asian Neolithic transition began ∼10,000 years ago^1^, was conquered by the Assyrians, Babylonians, Persians, and Romans, and was visited by the Egyptians and Greeks.^3^, ^4^, ^5^, ^6^ Among well-documented events within more recent historical times, three could potentially have involved significant immigration into the country. First, the Muslim expansion beginning in the 7^th^ century CE introduced the Islamic faith from its origin in the Arabian Peninsula.^7^ Second, in the 11^th^–13^th^ centuries CE, the Crusades resulted in the establishment of enclaves by substantial numbers of European Christians. 3, 4, 5, 7, 8 Finally, in the 16^th^ century CE, the Ottoman Empire expanded into this region and remained until the early part of the 20^th^ century.^3^ The current Lebanese population of almost four million people thus consists of a wide variety of ethnicities and religions, including Muslim, Christian, Druze, and others.

The Y chromosome carries the largest nonrecombining segment in the human genome, and consequently its haplotypes provide a rich source of information about male history.^9^ We set out to establish the extent of Y-chromosomal variation in Lebanon to determine whether this varies between subpopulations identified on the basis of geographical origin or religious affiliation and, if it does, to what extent such variation could be related to known historic or prehistoric events.

## Material and Methods

### Subjects and Comparative Datasets

We sampled 926 Lebanese men who had three generations of paternal ancestry in the country and who gave informed consent for this study, which was approved by the American University of Beirut IRB Committee. Each provided information on his geographical origin, classified into five regions: (1) Beirut (the capital city), (2) Mount Lebanon in the center, (3) the Bekaa Valley in the east, (4) the north, and (5) the south. Each also provided information on his religious affiliation: (1) Muslim, including the sects Shiite and Sunnite, (2) Christian, including the major sects Maronite, Orthodox, and Catholic, and (3) Druze, a distinct religion that has a 1000-year history and whose followers live mainly in Syria and Lebanon.

Comparative data on haplogroup frequencies were obtained from published sources and consenting individuals from the Genographic Public Participation dataset, whose participants can choose to make their data available for subsequent studies. For the Arabian Peninsula, published data from Omani Arabs^10^, Qatar, United Arab Emirates, and Yemen^11^ were used; in addition, we used data from the Genographic Public Participation dataset for individuals originating from Oman, Qatar, United Arab Emirates, Yemen, and Saudi Arabia (Table S2 in the Supplemental Data). Data from France^12^, Germany^13^, England^14^, and Italy^15^ were used to construct a representative western European sample as described below, and data from Turkey were also available.^16^

Combined Y-SNP plus Y-STR datasets were available from the Arabian Peninsula^10^, ^11^ and Turkey^16^. European data were extracted from the consented Genographic Project Public Participation database (Table S2).

### Historical Data

In addition to the contemporary subjects, we needed estimates of the likely genetic composition of the Crusaders. Historical sources^17^, ^18^, ^19^ show that four Crusades reached Lebanon—the first, second, third, and sixth—and that the main populations contributing were the French, Germans, English, and Italians; these sources suggest that the approximate numbers of men participating from the four countries were similar (Table 1). Y haplogroup frequencies are known in each of these modern populations^12^, ^13^, ^14^, ^15^, so if we assume that haplogroup frequencies were similar at the time of the Crusades, a weighted average western European haplogroup composition can be constructed (Table 2). This needed to be provided as numbers rather than frequencies for the tests described below. We therefore first scaled the total contribution from each country according to the smallest sample (the French^12^, n = 45) to produce the “weighted total” column in Table 2. We then divided each weighted total by the haplogroup frequency in that country to give a weighted number for each haplogroup from each country. Finally, we calculated the sum of these weighted numbers for each haplogroup and used the closest integer (bottom row in Table 2) in the analyses below.

**Table 1:** Numbers of Men Contributing to Each of the Crusades that Reached Lebanon According to Historical Sources^17^, ^18^, ^19^

Country	1st Crusade	2nd Crusade	3rd Crusade	6th Crusade	Total	Proportion
French	40,000	15,000	20,000	0	75,000	0.28
German	23,000	15,000	1,000	25,000	64,000	0.24
English	23,000	15,000	30,000	0	68,000	0.26
Italian	59,000	0	0	0	59,000	0.22
Total	145,000	45,000	51,000	25,000	266,000	1.00

**Table 2:** Construction of a Western European Y Haplogroup Sample Weighted According to the Relative Contribution from Each Country

	E3b	G	I	J^∗^(xJ2)	J2	K2	L	R1b	Other	Total	Weighted total
European Y-Chromosomal Haplogroup Numbers from Previous Studies											

French^12^	2	0	6	-	4	0	0	31	2	45	45
Germans^13^	75	-a	287	-	49	-	-	473	331	1215	38.4
English^14^	24	-	163	3	25	-	-	616	45	876	40.8
Italians^15^	88	75	52	14	140	-	-	280	50	699	35.4
											159.6

Weighted Numbers Used											

French	2	0	6	0	4	0	0	31	2	45	
German	2.4	0	9.1	0	1.5	0	0	14.9	10.5	38.4	
English	1.1	0	7.6	0.1	1.2	0	0	28.7	2.1	40.8	
Italy	4.5	3.8	2.6	0.7	7.1	0	0	14.2	2.5	35.4	
Western European combined	9.9	3.8	25.3	0.8	13.8	0	0	88.8	17.1	159.6	
Western European (integer)	10	4	25	1	14	0	0	89	17	160	

a Rare haplogroup not typed in the relevant study; value set to zero.

### Genotyping

**Figure 2:**
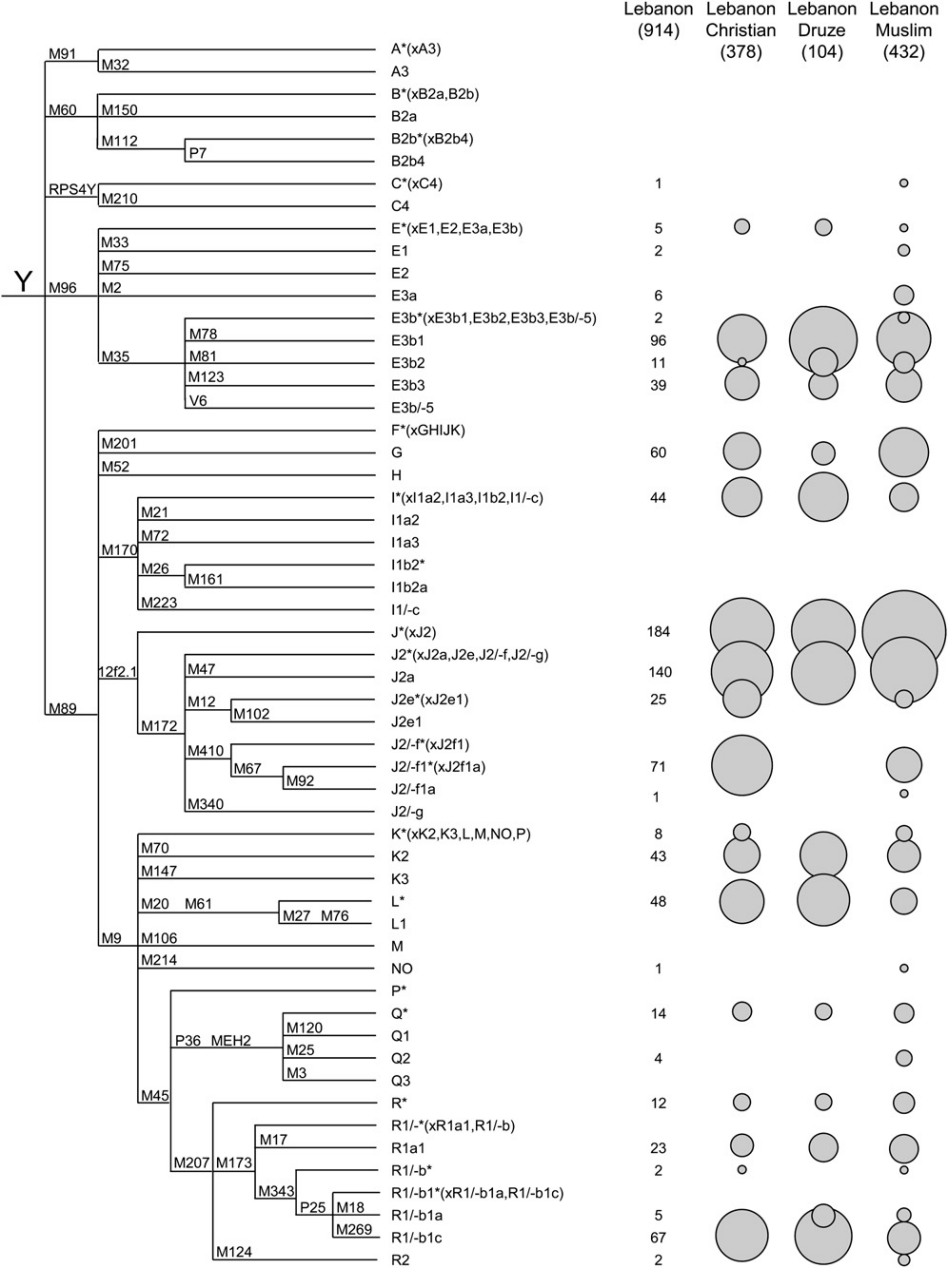
Y-Chromosomal Haplogroups Identified in Lebanese Subpopulations The phylogenetic tree defined by the markers used is shown on the left, and the haplogroup names are given in the middle. Nomenclature is based on the 2003 YCC tree^9^, with departures indicated by “/-”. The absolute number of chromosomes within each haplogroup in the entire sample is shown in the “Lebanon” column, and the relative frequency within each of the three religious groups is shown on the right by the relative sizes of the circles.

Samples were genotyped with a set of 58 Y-chromosomal binary markers by standard methods^20^ (Figure 2). These markers define 53 haplogroups (including paragroups), 27 of which were present in the Lebanese sample. We also typed a subset (the first 587 individuals collected, and thus with unbiased ascertainment) with 11 Y-STRs by using standard methods^21^, ^22^ (Table S1). STR alleles were named according to current recommendations^23^, except that “*389b*” was used in place of “*DYS389II*”; *398b* = (*DYS389II* − *DYS389I*).

### General Statistical Analyses

Analysis of molecular variance (AMOVA)^24^, population pairwise genetic distances, and Mantel tests^25^ were performed with the package Arlequin 3.11.^26^ Admixture analyses were carried out with Admix2_0.^27^ Median-joining networks^28^ were calculated with Network 4.2 (Fluxus-Engineering). Such networks were highly reticulated, and we reduced reticulations by first weighting the loci according to the inverse of their variance in the dataset used^29^ and subsequently constructing a reduced-median network^30^ to form the input of the median-joining network. Male effective population sizes were calculated with BATWING^31^ with a demographic model that assumed a period of constant size followed by exponential growth; prior values were set for other parameters as described previously.^20^

### Computation of Drift Probabilities

We wished to calculate the probability that a haplotype could increase from a deduced initial frequency to an observed current frequency by chance over a period specified by the historical record. In addition, we wished to evaluate the influence that admixture with an outside population might have on this probability. We had detailed data consisting of Y-SNP and Y-STR sets for some relevant groups and relied upon the YHRD database for data from other populations. A number of applications are available for estimating migration rates; these applications account for coalescence, mutation, and migration, including estimates of variation of migration, over a period of time.^32^, ^33^, ^34^, ^35^, ^36^, ^37^, ^38^ However, none of the packages address the specific question of testing whether drift alone could reasonably account for the emergence of modern levels of haplogroup or haplotype frequencies in the population or how much migration for a specified epoch could affect these rates if the available historical information is incorporated. We have therefore chosen to directly employ a Wright-Fisher model with sampled migration to compute the effects of drift given an admixture event of known duration.

The Wright-Fisher model^39^, ^40^ entirely replaces each generation with each succeeding one. The offspring select their parents randomly. The following calculation outlines the Wright-Fisher drift model, describing how the probability of seeing some particular number of members of a population carrying a haplotype will evolve over time. Then it considers the following circumstance: Two populations are evolving according to the Wright-Fisher model and the island model of Haldane^41^. First, a European population carrying a particular haplotype of interest described below (Western European Specific 1, WES1) experiences drift freely. Over some period of time, some number of this population is selected randomly and travels to Lebanon. Each generation, the children randomly select their parents from the mixed Lebanese and migrant European populations.

Given that a proportion *p* parents are of some particular haplotype, the probability that the selected number *X*(*t* + 1) of *l* children out of an effective population of size *N* is $P\left(X\left(t+1\right)=l\right)=\left(\begin{array}{c} N\\ l \end{array}\right)p^{l}{\left(1-p\right)}^{N-l}$. Given that *j* out of *N* parents are of the haplotype of interest, then *p* = *j/N*. Therefore, the probability of finding *l* children of the haplotype of interest given *j* parents is $P\left(X\left(t+1\right)=l|X\left(t\right)=j\right)=\left(\begin{array}{c} N\\ l \end{array}\right){\left(\frac{j}{N}\right)}^{l}{\left(1-\frac{j}{N}\right)}^{N-l}$.

Given a distribution of probabilities *P*(*X*(*t*) *= j*) of finding *j* children of the haplotype of interest at some generation *t*, the probability *P*(*X*(*t* + *1*) *= l*) of finding *l* of the haplotype at time *t* + 1 is $P\left(X\left(t+1\right)=l\right)=\sum_{j=0}^{N}P\left(X\left(t+1\right)=l|X\left(t\right)=j\right)P\left(X\left(t\right)=j\right)$. The chances *p_f_* of finding at least some fraction *f* of that haplotype after *t* = *T* generations is $p_{f}=\sum_{j\geq f\cdot N}P\left(X\left(T\right)=j\right)$.

We can extend the above argument to include the admixture of one population with another if we replace the population sampled by the children with an expanded pool that includes contributions from the incoming population. In this case, a population labeled *W* carrying among them members of the WES1 haplotype mixes with a native Lebanese population labeled *L*. Given an effective population *N_L_* of Lebanese Christians and an effective population *N_W_* of Europeans, the fraction of migrants from which the next generation can choose will be $m=\frac{N_{W}}{N_{L}+N_{W}}$. The fraction of Lebanese Christians bearing the WES1 marker will be $p_{L}=\frac{j_{L}}{N_{L}}$, and that of Europeans will be $p_{W}=\frac{j_{W}}{N_{W}}$. The total admixed fraction of WES1 presented to the next generation will be $p_{A}\left(j_{L},j_{W}\right)=\left(1-m\right)p_{L}+mp_{W}=\frac{j_{L}+j_{W}}{N_{L}+N_{W}}$.

The number of WES1 individuals, *j_W_*, that traveled to Lebanon is a random variable *X_W_*(*t*) that will have a distribution determined by sampling *N_W_* admixing WES1 members from the European population, which itself is experiencing drift with probability *P*(*X_E_*(*t*) = *j_E_*) in an effective European population *N_E_*. Therefore, the distribution of *j_W_* will be determined by $P\left(X_{W}\left(t\right)=j_{W}\right)=\sum_{j_{E}=0}^{N_{E}}\left(\begin{array}{c} N_{W}\\ j_{W} \end{array}\right){\left(\frac{j_{E}}{N_{E}}\right)}^{j_{W}}{\left(1-\frac{j_{E}}{N_{E}}\right)}^{N_{W}-j_{W}}P\left(X_{E}\left(t\right)=j_{E}\right)$. Then the admixed probability $P\left(X_{L}\left(t+1\right)=l|X_{L}\left(t\right)=j_{L},X_{W}\left(t\right)=j_{W}\right)$ that *l* children will have selected WES1 parents from *N_L_* Lebanese and *N_W_* WES1 parents is $P\left(X_{L}\left(t+1\right)=l|X_{L}\left(t\right)=j_{L},X_{W}\left(t\right)=j_{W}\right)=\left(\begin{array}{c} N_{L}\\ l \end{array}\right){\left(p_{A}\left(j_{L},j_{W}\right)\right)}^{l}{\left(1-p_{A}\left(j_{L},j_{W}\right)\right)}^{N_{L}-l}$. If we sum over the distributions of *j_L_* and *j_L_*, the final probability distribution of possible future selections of WES1 by the children will be $P\left(X_{L}\left(t+1\right)=l\right)=\sum_{j_{L}=0}^{N_{L}}\sum_{j_{W}=0}^{N_{W}}\{P\left(X_{L}\left(t+1\right)=l|X_{L}\left(t\right)=j_{L},X_{W}\left(t\right)=j_{W}\right)\times P\left(X_{L}\left(t\right)=j_{L}\right)P\left(X_{W}\left(t\right)=j_{W}\right)\}$. The initial condition of finding *p_0_* assumed as an initial Lebanese fraction of the WES1 marker is specified by requiring $P\left(j,0\right)=\{\begin{array}{c} 1\text{where}j=\left\lfloor p_{0}N\right\rfloor \\ 0\text{elsewhere} \end{array}$.

Computations were performed in C++ with the binomial distribution function implemented in the Gnu Scientific Library.^42^

## Results

### Genetic Structure within Lebanon

The Lebanese sample was subdivided geographically into five subpopulations: one from the capital city, Beirut, and four from other geographically distinct regions that included the Bekaa in the east, the north, the south, and the central Mount Lebanon. After excluding the Beirut individuals because of their diverse recent origins, we estimated the proportions of variation within and between the geographical subpopulations on the basis of the haplogroup frequencies (Table 3). Even within this small geographical area, a highly significant proportion of the variation (0.39%, p < 0.01) was found between the regions, a conclusion reinforced by the finding that genetic distances were significantly greater than zero between several of the pairs of subpopulations when either Y-SNPs or Y-STRs were used (Table 4). The total Lebanese sample could also be subdivided according to religion (Muslim, Christian, or Druze) or religious sect (Shiite, Sunnite, Maronite, or Druze). Using these categories, we found that the proportion of variation between the subpopulations was more than three times higher (1.42%, 1.32%, both p < 0.01; Table 3) than between the geographic regions. Again, many of the genetic distances between religious groups or sects were significant (Table 4). The divisions are not independent because the religious communities show geographical clustering, and when allowance was made for religious affiliation (Muslim, Christian, Druze), a Mantel test^25^ showed that no additional variation was explained by geographical factors (the four regions).

**Table 3:** Variation in Y-Chromosomal Haplogroup Frequencies between Subpopulations within Lebanon

Basis of Division	Populations	Percentage of Variation	
		Within Populations	Among Populations
Geography	Bekaa, Mt. Lebanon, North, South	99.61	0.39^a^
Religious affiliation	Muslim, Christian, Druze	98.58	1.42^a^
Sect	Shiite, Sunnite, Maronite, Druze	98.68	1.32^a^

Variation was determined by an analysis of molecular variance.

a p < 0.01.

**Table 4:** Pairwise Genetic Distances between Lebanese Subpopulations

Pairwise F_ST_ (SNPs)					
Geographical region		Beirut	Bekaa	Mt. Lebanon	North
	Bekaa	−0.0028			
	Mt. Lebanon	0.0075^b^	0.0012		
	North	0.0086^b^	0.0004	0.0033^b^	
	South	−0.0020	−0.0029	0.0101^b^	0.0047^b^

Religion		Christian	Druze		
	Druze	0.0117^b^			
	Muslim	0.0147^b^	0.0145^b^		

Sect		Druze	Maronite	Shiite	
	Maronite	0.0166^b^			
	Shiite	0.0186^b^	0.0195^b^		
	Sunnite	0.0115^b^	0.0145^b^	0.0000	

Pairwise Φ_ST_ (STRs)					

Geographical region		Beirut	Bekaa	Mt. Lebanon	North
	Bekaa	0.0071			
	Mt. Lebanon	0.0099^a^	0.0056		
	North	0.0063	0.0037	0.0042	
	South	0.0001	0.0001	0.0081^a^	0.0061^a^

Religion		Christian	Druze		
	Druze	0.0060			
	Muslim	0.0117^a^	0.0073		

Sect		Druze	Maronite	Shiite	
	Maronite	0.0041			
	Shiite	0.0071	0.0179^b^		
	Sunnite	0.0134	0.0133^b^	−0.0001	

a p < 0.05.

b p < 0.01.

### Identification of Potential Sources for Lebanese Genetic Structure

Because religious affiliation has the greatest impact on the patterns of genetic variation in Lebanese populations, and because these religions have originated within historical times, we first sought explanations for the genetic differences from the documented historical migrations: Muslim, Crusader, and Ottoman (Figure 1). Using historical evidence, we identified source regions for these migrations in the Arabian Peninsula, western Europe, and Turkey, respectively. We then collected suitable Y-chromosomal SNP datasets from these areas. For the Arabian Peninsula and Turkey this was simple, and data from France, Germany, England, and Italy^15^ were used to construct a suitable western European sample as described in the Material and Methods section. Because we needed to compare the Lebanese data with the same haplogroups in these additional datasets, we combined some related haplogroups to form eight haplogroups [E3b, G, I, J^∗^(xJ2), J2, K2, L, and R1b] that were each present in Lebanon at > 4%, together accounted for 90% of the Lebanese sample, and could be compared with the categories used by other authors (Table 5).

**Table 5:** Haplogroup Fequencies in Lebanon and Potential Source Populations

	E3b	G	I	J^∗^(xJ2)	J2	K2	L	R1b	Other	Total
Lebanon (number)	148	60	44	184	237	43	48	74	97	935
Lebanon (frequency)	0.158	0.064	0.047	0.197	0.253	0.046	0.051	0.079	0.104	
Arabian Peninsula (number)	51	12	0	196	43	18	8	9	96	433
Arabian Peninsula (frequency)	0.118	0.028	0.000	0.453	0.099	0.042	0.018	0.021	0.222	
p value Arabian Peninsula v Lebanon	0.0481	0.0049	0.0000	0.0000^a^	0.0000	0.7126	0.0043	0.0000		
Western Europeans (estimated number)	10	4	25	1	14	0	0	89	17	160
Western Europeans (estimated frequency)	0.063	0.025	0.156	0.006	0.088	0.000	0.000	0.556	0.106	
p value W. Europeans vs. Lebanon	0.0014	0.0274	0.0000^a^	0.0000	0.0000	0.0056	0.0033	0.0000^a^		
Turkey (number)	56	57	28	48	127	13	22	83	89	523
Turkey (frequency)	0.107	0.109	0.054	0.092	0.243	0.025	0.042	0.159	0.170	
p value Turkey vs. Lebanon	0.0068	0.0025	0.5839	0.0000	0.6523	0.0440	0.4270	0.0000^a^		

a Significantly higher in source after Bonferroni correction.

A standard approach to determining whether migration from these countries might have contributed to the Lebanese population would be to perform an admixture analysis with the putative source as one parental population. Taking such an approach, we could identify possible contributions from the Arabian Peninsula to Lebanese Muslims and from western Europe to Lebanese Christians, but the uncertainties in the estimates were large, and no meaningful result was obtained when Turkey was used as a potential source (Table 6). In order to investigate further, we then compared individual haplogroup frequencies in Lebanon and the putative source regions, and we identified haplogroups that differed significantly in frequency by using a Chi-square test with a Bonferroni correction for multiple testing. A number of haplogroups were found at significantly higher frequency in the potential source region than in Lebanon: J^∗^(xJ2) in the Arabian Peninsula, I and R1b in the western European sample, and R1b in Turkey (Table 5). Because the extent to which the western European sample used here might represent the Crusaders is uncertain, we investigated the sensitivity of our conclusion to the composition of this sample. Haplogroups I and R1b were both present at higher frequency in each of the individual populations, and the difference was significant for R1b in all four populations and for I in two of them (Germans and English). No other haplogroup was at a significantly higher frequency in any of the individual populations than in Lebanon. We therefore conclude that this is a robust finding.

**Table 6:** Admixture Analyses

Parental 1	Parental 2	Admixed	Parental 1 Contribution
Arabian Peninsula	Lebanese non-Muslims	Lebanese Muslims	37%, SD 11%
Western Europe	Lebanese non-Christians	Lebanese Christians	10%, SD 7%
Turkey	Lebanese non-Muslims	Lebanese Muslims	38%, SD 68%

These observations, together with the historical information, led us to formulate three specific hypotheses: that many J^∗^(xJ2) chromosomes were introduced into Lebanese Muslims by the Muslim expansion from the Arabian Peninsula; that some I and R1b chromosomes were introduced into Lebanese Christians by immigrating European Christians, perhaps during the time of the Crusades; and that additional R1b chromosomes were introduced into Lebanese Muslims during the Ottoman expansion. We do not, of course, imply that these migrations carried only these haplogroups; obviously, they would have involved populations containing multiple haplogroups. The signal of migration, however, should be most readily detected in the highly differentiated haplogroups. J^∗^(xJ2) was found to be much more frequent in Lebanese Muslims than in Lebanese non-Muslims (25% vs. 15%, p < 0.0001). The combined I + R1b frequency was higher in Lebanese Christians than in Lebanese non-Christians (16% vs. 10%, p = 0.01), as were both of the individual haplogroups (I: 5.8% vs. 4.0%, p = 0.21; R1b 10% vs. 6.3%, p = 0.03), although the difference for haplogroup I alone did not reach statistical significance. The R1b frequency was, however, significantly *lower* in Lebanese Muslims than in Lebanese non-Muslims (4.7% vs. 11%, p = 0.0005). The hypotheses of male-mediated gene flow accompanying the earlier Muslim and Crusader migrations are therefore supported, but our data provide no evidence for a differential genetic impact of the Ottoman expansion.

### Evidence for Migration from Haplotype Structure

**Figure 3:**
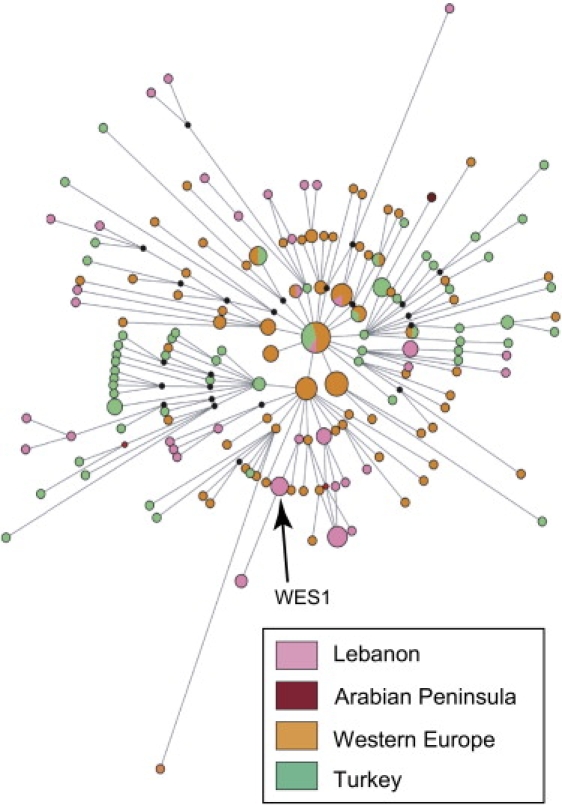
Network of STR Variation within Haplogroup R1b Circles represent haplotypes defined by nine STRs; area is proportional to frequency, and color indicates the region of origin. Lines represent the mutational differences between haplotypes.

Finally, we investigated the possible origins of the J^∗^(xJ2), I, and R1b chromosomes in more detail by using information from the STR haplotypes. We visualized STR haplotypes within each haplogroup by using networks^28^ constructed with the nine Y-STRs common to all datasets. Geographical structure was seen in the I and R1b networks (Figure 3), but not in the J^∗^(xJ2) network. The geographical distributions of Lebanese haplotypes were then investigated in the Y chromosome Haplotype Reference Database^43^ (YHRD, release 21) with seven Y-STRs so that 51,253 entries from 447 populations could be interrogated. Of the 30 Lebanese R1b haplotypes, six (representing seven individuals) were absent from the database, and 22 of the remaining 24 showed distributions that included Europe and western Asia, as would generally be expected. Most of these haplotypes thus did not provide more precise subregional information about their likely place of origin.

**Figure 4:**
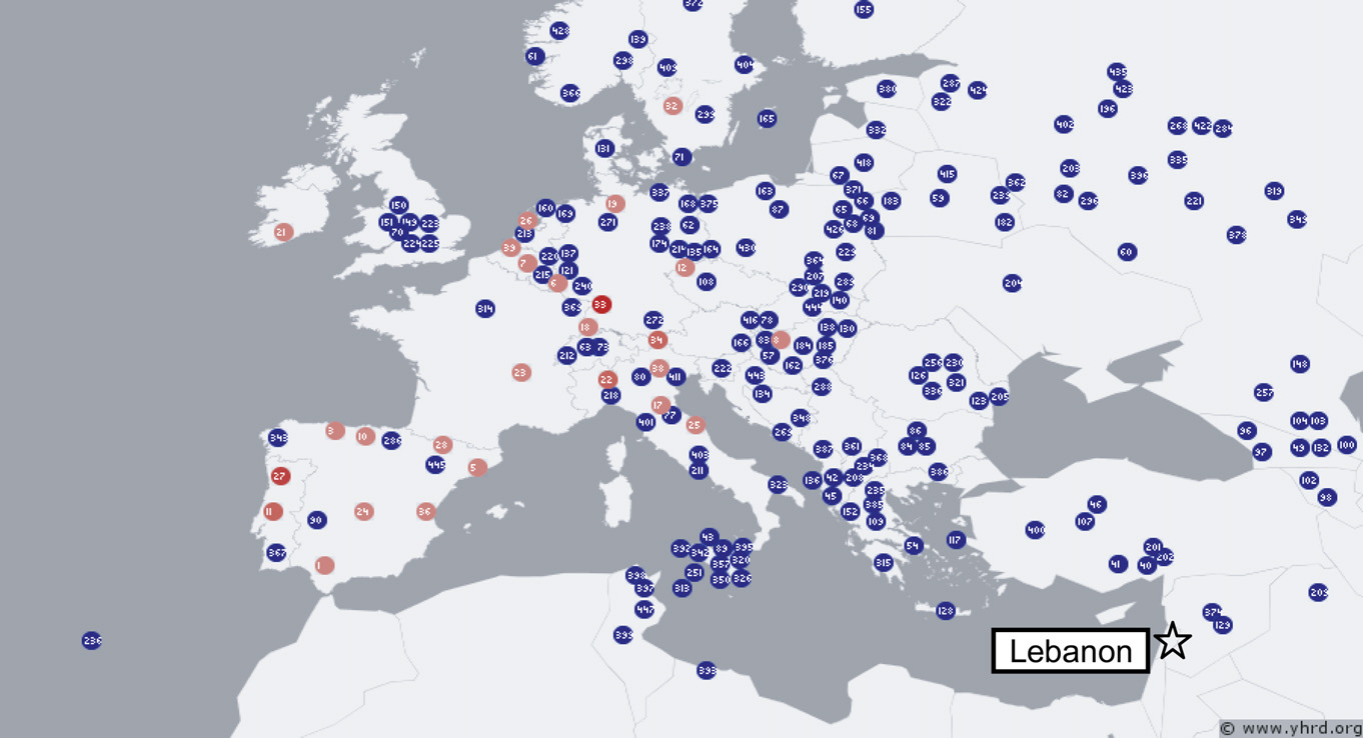
Geographical Distribution of WES1, the Most Common R1b Haplotype in Lebanese Christians This haplotype is *DYS19, DYS389I, DYS389b, DYS390, DYS391, DYS392, DYS393* 14, 12, 16, 24, 10, 13, 13. Population samples containing the haplotype are shown in red, and those lacking it are shown in blue. Note the highly specific western European distribution and the absence of the haplotype from populations near Lebanon. Data are from YHRD.

One haplotype (WES1, Western European Specific 1), however, stood out for two reasons. First, it showed a common but strictly western European distribution among the indigenous populations in the YHRD; it was present in 26/81 European populations west of Hungary and in zero populations east of this longitude (Figure 4). Second, and in contrast to its distribution in the database, it was the most common R1b haplotype in the Lebanese Christians tested (5/27, 19% of R1b, or about 2% of the total Lebanese Christian haplotypes).

Because this Lebanese occurrence lies far outside the normal range of this haplotype, we investigated how likely a haplotype was to rise to this frequency by chance. The first test considered the chances of observing modern levels of the WES1 haplotype among Lebanese Christians without any migration. No WES1 members were found in >1,000 Middle Eastern individuals in the YHRD. Making the highly conservative assumption that its frequency *p_0_* in the Middle East outside the Lebanese Christians was ∼0.1% (the maximum observed size consistent with zero in the sample) and a male effective population size of *N_L_* ≈1000 for the Lebanese Christians estimated from our data with BATWING, we calculated the probability of observing the modern fraction *f* of 2% or more as <0.02 (Material and Methods). In contrast, given an input of western Europeans, selected from an evolving effective population *N_E_* ≈5000, who were carrying WES1 at 0.21% (the weighted average of the YHRD frequencies from England, France, Germany, and Italy), the probability of reaching 2% or more among Lebanese Christians exceeded 0.05 for an admixing population fraction *m* of ∼10.6% or greater (Table 7). It has been assumed that a total of 32 generations have passed since the start of the admixture event^44^, with mixing only during the first seven generations. Thus, WES1 is likely to have originated in western Europe and shows exactly the pattern expected for a European lineage introduced by the Crusaders.

**Table 7:** Estimated Influence of Historical Western European Admixture on the Frequency of WES1 in Modern Lebanese Christians

*m*^a^	*P*(*l* ≥ *0.02* × *N_L_*)^b^	*P*(*l* = 0)^c^
0	0.0189	0.9425
0.0500	0.0325	0.9001
0.1000	0.0482	0.8545
0.1055	0.0500	0.8492
0.1500	0.0656	0.8069
0.2000	0.0857	0.7561
0.3000	0.1347	0.6465
0.4000	0.1998	0.5258
0.5000	0.2889	0.3949

a Level of admixture of a western European population (*N_W_* = 5,000) carrying WES1 at 0.21% for seven generations to a Lebanese Christian population (*N_L_* = 1,000) carrying WES1 at 0.01%.

b Probability that WES1 would have reached 2% or more after 32 generations.

c Probability that WES1 would have been extirpated after 32 generations.

Likewise, one can test the question of whether the difference in J^∗^(xJ2) frequencies between Muslims (25%) and non-Muslims (15%) would have emerged by drift without enhancement during the Islamic expansion from the Arabian Peninsula by considering the probability that the 15% frequency could have drifted up to 25% or more by chance in the ∼42 generations since the Islamic expansion. For an assumed effective population size of ∼5,000, this is 0.0023, and thus, again, admixture seems likely to have contributed.

## Discussion

We find a striking correspondence between documented historical migrations to Lebanon and current patterns of genetic variation within the country. The variation was perhaps initially low or structured by geography but was subsequently accentuated by religion-driven migration into specific communities within Lebanon. Two of the three major migrations have left a detectable impact, and conversely, the main features of the differentiation within Lebanon can be accounted for by these events. It is likely that earlier migratory events have also contributed to the genetic diversity in present-day Lebanese populations, but because these migrations would have occurred before the present religious affiliations and communities were created, they are expected to have shaped the genetic makeup of the country as a whole rather than specific religious subpopulations.

Genetic structuring by religion has been rarely reported in human populations: it was not detectable, for example, among Muslim and Hindu paternal^45^ or maternal^46^ lineages in India. A Y-chromosomal lineage that is rare in India but common in western Asia was found at unusually high frequency in an Indian Shiya Muslim sample^47^, and structuring by religion has been seen among Jewish maternal (although not paternal) lineages^48^. Such structure might only arise when several unusual criteria are met: migrations based on religion must take place between areas with different representative Y-chromosomal types, and they must establish genetically differentiated communities that remain stable over long time periods. In Lebanon, these conditions appear to have been met for over 1,300 years.

## Web Resources

The URLs for data presented herein are as follows:

Arlequin, http://lgb.unige.ch/arlequin/
Genographic Project, https://www.nationalgeographic.com/genographic/index.html
Network, http://www.fluxus-engineering.com/sharenet.htm
Y Chromosome Haplotype Reference Database (YHRD), http://www.yhrd.org/index.html

## Supplemental Data

Document S1. Two Tables
