# Supplementary material for: Y-Chromosomal Diversity in Lebanon Is Structured by Recent Historical Events
Source: Am J Hum Genet. 2008 Apr 4;82(4):873–82. doi: 10.1016/j.ajhg.2008.01.020 (PMC2427286; doi:10.1016/j.ajhg.2008.01.020)
Supplement: Document S1. Two Tables [file mmc1.pdf]

The American Journal of Human Genetics, April 2008

Supplemental Data

Y-Chromosomal Diversity in Lebanon

Is Structured by Recent Historical Events

Pierre A. Zalloua, Yali Xue, Jade Khalife, Nadine Makhoul, Labib Debiane, Daniel E. Platt, Ajay K. Royyuru, Rene J. Herrera, David F. Soria Hernanz, Jason Blue-Smith, R. Spencer Wells, David Comas, Jaume Bertranpetit, Chris Tyler-Smith, and The Genographic Consortium

The Genographic Consortium: Theodore G. Schurr, University of Pennsylvania, Philadelphia, PA, USA; Fabrício R. Santos, Universidade Federal de Minas Gerais, Belo Horizonte, Minas Gerais, Brazil; Lluis Quintana-Murci, Institut Pasteur, Institut Pasteur, Paris, France; Jaume Bertranpetit, Universitat Pompeu Fabra, Barcelona, Catalonia, Spain; David Comas, Universitat Pompeu Fabra, Barcelona, Catalonia, Spain; Chris Tyler-Smith, The Wellcome Trust Sanger Institute, Hinxton, UK; Pierre A. Zalloua, Lebanese American University, Chouran, Beirut, Lebanon; Elena Balanovska, Russian Academy of Medical Sciences, Moscow, Russia; Oleg Balanovsky, Russian Academy of Medical Sciences, Moscow, Russia; Doron M. Behar, Genomics Research Center, Family Tree DNA, Houston, TX, USA; R. John Mitchell, La Trobe University, Melbourne, Victoria, Australia; Li Jin, Fudan University, Shanghai, China; Himla Soodyall, National Health Laboratory Service, Johannesburg, South Africa; Ramasamy Pitchappan, Madurai Kamaraj University, Madurai, Tamil Nadu, India; Alan Cooper, University of Adelaide, South Australia, Australia; Ajay K. Royyuru, IBM, Yorktown Heights, NY, USA; Saharon Rosset, IBM T. J. Watson Research Center, Yorktown Heights, NY, USA; Jason Blue-Smith, David F. Soria Hernanz, and R. Spencer Wells, National Geographic Society, Washington, DC, United States of America.

Table S1. Distribution of Y-chromosomal haplogroups and haplotypes among different geographical regions and religious affiliations in Lebanon

**No.**	**Haplogroup**	**Allele status at**											**Origin**	**Sect**	**Religion**
		**DYS 19**	**DYS 388**	**DYS 389I**	**DYS 389b**	**DYS 390**	**DYS 391**	**DYS 392**	**DYS 393**	**DYS 437**	**DYS 438**	**DYS 439**			
1	C*(xC4)	17	13	15	16	24	9	12	13	14	10	11	Bekaa	Shiite	Muslim
2	E*(xE1,E2,E3a,E3b)	14	13	13	17	24	10	11	13	14	10	12	Beirut	Catholic	Christian
3	E*(xE1,E2,E3a,E3b)	13	12	13	17	24	10	11	14	14	10	12	Mt Leb	Orthodox	Christian
4	E*(xE1,E2,E3a,E3b)	13	12	13	18	25	10	11	13	14	10	13	South	Shiite	Muslim
5	E1	14	12	12	18	22	9	12	13	16	10	10	South	Shiite	Muslim
6	E3a	15	12	13	18	21	10	13	13	14	9	12	South	Shiite	Muslim
7	E3a	15	12	13	17	21	10	11	15	14	11	13	South	Shiite	Muslim
8	E3a	15	12	13	17	21	10	11	13	14	12	12	South	Shiite	Muslim
9	E3a	14	12	13	17	21	10	11	13	14	11	11	South	Shiite	Muslim
10	E3a	15	12	14	17	21	10	11	13	14	11	12	Beirut	Sunnite	Muslim
11	E3b*(xE3b1,E3b2,E3b3,E3b/-5)	14	12	13	16	23	9	13	14	14	11	11	South	Shiite	Muslim
12	E3b*(xE3b1,E3b2,E3b3,E3b/-5)	17	13	14	16	23	9	11	13	14	10	11	Bekaa	Shiite	Muslim
13	E3b1	14	12	13	18	25	10	11	13	14	10	12	Mt Leb	Catholic	Christian
14	E3b1	13	12	13	17	23	10	11	13	14	10	12	Beirut	Druze	Druze
15	E3b1	13	12	13	17	24	10	11	13	14	10	13	Mt Leb	Druze	Druze
16	E3b1	14	12	12	18	25	11	11	13	14	10	11	Mt Leb	Druze	Druze
17	E3b1	15	12	13	16	24	10	11	13	14	10	11	Beirut	Latin	Christian
18	E3b1	14	13	13	19	24	10	11	13	14	10	13	North	Maronite	Christian

19	E3b1	14	12	12	17	24	10	11	13	14	11	11	North	Maronite	Christian
20	E3b1	13	12	12	19	25	10	12	13	14	10	12	Mt Leb	Maronite	Christian
21	E3b1	14	12	13	17	24	11	11	13	14	10	12	Mt Leb	Maronite	Christian
22	E3b1	13	12	14	17	24	10	11	13	14	10	12	North	Maronite	Christian
23	E3b1	13	12	12	17	23	10	11	13	14	10	12	Bekaa	Maronite	Christian
24	E3b1	14	13	13	18	24	10	11	13	14	10	13	Mt Leb	Maronite	Christian
25	E3b1	13	12	13	17	23	10	11	13	14	11	11	North	Maronite	Christian
26	E3b1	13	12	14	17	24	10	11	14	14	10	12	North	Maronite	Christian
27	E3b1	14	12	13	17	24	11	11	13	14	10	12	Mt Leb	Maronite	Christian
28	E3b1	13	12	13	17	24	11	11	12	14	10	12	North	Maronite	Christian
29	E3b1	14	12	13	17	24	11	12	13	14	10	11	North	Maronite	Christian
30	E3b1	13	12	14	17	24	10	11	14	14	10	12	North	Orthodox	Christian
31	E3b1	14	12	13	17	24	10	12	13	14	10	12	North	Orthodox	Christian
32	E3b1	14	12	14	17	24	10	13	13	14	11	11	North	Orthodox	Christian
33	E3b1	13	12	13	18	23	10	11	13	14	10	12	Bekaa	Orthodox	Christian
34	E3b1	13	12	13	18	23	10	11	13	14	10	11	South	Shiite	Muslim
35	E3b1	13	12	13	17	23	10	11	13	14	11	11	Mt Leb	Shiite	Muslim
36	E3b1	13	12	13	17	24	10	11	13	14	10	13	Mt Leb	Shiite	Muslim
37	E3b1	13	12	13	17	23	10	11	13	14	11	11	North	Shiite	Muslim
38	E3b1	14	12	13	16	24	10	11	12	14	8	10	Bekaa	Shiite	Muslim
39	E3b1	14	12	13	17	24	10	11	13	14	10	12	Mt Leb	Shiite	Muslim
40	E3b1	14	13	12	17	24	10	11	14	14	10	13	South	Shiite	Muslim
41	E3b1	13	12	13	17	23	10	11	13	14	10	11	South	Shiite	Muslim
42	E3b1	14	12	13	17	23	10	11	13	14	10	11	South	Shiite	Muslim
43	E3b1	14	12	13	17	24	11	12	12	14	10	11	Bekaa	Shiite	Muslim
44	E3b1	14	12	13	17	23	10	11	13	14	10	12	South	Shiite	Muslim
45	E3b1	13	12	13	17	23	10	10	13	14	11	11	Bekaa	Shiite	Muslim
46	E3b1	13	12	12	17	25	10	11	13	14	10	10	Bekaa	Shiite	Muslim
47	E3b1	13	12	13	18	23	10	11	13	14	10	11	South	Shiite	Muslim
48	E3b1	14	12	13	17	24	11	12	12	14	10	11	Bekaa	Shiite	Muslim
49	E3b1	14	12	13	17	24	10	12	12	14	10	11	North	Shiite	Muslim
50	E3b1	13	12	13	17	24	10	11	13	14	10	13	South	Shiite	Muslim
51	E3b1	13	14	13	17	24	10	11	13	14	10	12	Beirut	Sunnite	Muslim
52	E3b1	15	12	14	17	24	10	11	13	14	10	11	Beirut	Sunnite	Muslim
53	E3b1	14	12	13	17	24	10	11	13	14	10	11	North	Sunnite	Muslim
54	E3b1	13	12	12	19	24	10	11	13	14	10	12	North	Sunnite	Muslim
55	E3b1	14	12	13	17	24	11	12	13	14	10	11	North	Sunnite	Muslim
56	E3b1	14	12	13	17	24	10	11	13	14	10	12	Beirut	Sunnite	Muslim
57	E3b1	13	12	13	17	23	10	12	13	14	10	13	Mt Leb	Sunnite	Muslim
58	E3b1	13	12	13	17	23	10	12	13	14	10	12	Mt Leb	Sunnite	Muslim
59	E3b1	13	12	13	17	24	10	11	13	14	10	12	Bekaa	Sunnite	Muslim
60	E3b1	16	12	13	16	23	10	11	13	14	10	11	South	Sunnite	Muslim
61	E3b1	13	12	12	17	24	10	11	14	15	11	12	North	Sunnite	Muslim
62	E3b1	13	12	13	17	24	10	11	13	14	10	12	Beirut	Sunnite	Muslim
63	E3b1	13	12	13	17	23	10	12	13	14	10	12	Mt Leb	Sunnite	Muslim
64	E3b1	13	12	14	17	23	10	11	13	14	10	12	Beirut	Sunnite	Muslim
65	E3b1	11	12	13	17	23	10	12	13	14	10	12	South	Sunnite	Muslim
66	E3b2	13	12	13	16	24	9	11	13	14	10	11	North	Maronite	Christian
67	E3b2	13	12	13	16	24	9	11	13	14	10	10	South	Shiite	Muslim
68	E3b2	13	12	13	16	24	9	11	13	14	10	10	South	Shiite	Muslim
69	E3b2	13	12	13	16	24	9	11	13	14	10	10	Mt Leb	Sunnite	Muslim
70	E3b2	14	12	14	16	24	9	11	13	14	10	10	North	Sunnite	Muslim
71	E3b2	14	12	14	16	24	10	11	13	14	10	10	Mt Leb	Sunnite	Muslim

72	E3b2	14	12	14	16	24	9	11	13	14	10	10	Beirut	Sunnite	Muslim
73	E3b3	13	12	13	19	24	10	12	13	14	10	10	Mt Leb	Catholic	Christian
74	E3b3	13	12	13	18	23	10	11	13	14	10	13	Mt Leb	Druze	Druze
75	E3b3	13	12	13	18	24	10	11	13	14	10	12	Mt Leb	Druze	Druze
76	E3b3	13	12	13	18	23	10	11	13	14	10	13	Bekaa	Maronite	Christian
77	E3b3	13	12	13	20	23	11	11	13	14	10	13	North	Maronite	Christian
78	E3b3	13	12	12	18	23	10	11	13	14	10	13	North	Maronite	Christian
79	E3b3	13	12	13	18	25	10	11	13	14	10	12	Mt Leb	Maronite	Christian
80	E3b3	13	12	12	18	23	10	11	13	14	10	13	Bekaa	Maronite	Christian
81	E3b3	13	12	13	19	25	10	11	13	14	10	12	Mt Leb	Maronite	Christian
82	E3b3	13	12	13	18	24	10	11	13	14	10	12	North	Maronite	Christian
83	E3b3	13	12	13	18	24	10	11	13	14	10	12	North	Maronite	Christian
84	E3b3	14	12	14	19	23	9	11	14	14	11	12	Beirut	Orthodox	Christian
85	E3b3	13	12	13	18	25	10	11	13	14	11	12	North	Orthodox	Christian
86	E3b3	13	12	13	17	24	10	11	13	14	10	13	Bekaa	Shiite	Muslim
87	E3b3	13	12	12	19	24	10	11	13	14	10	12	South	Shiite	Muslim
88	E3b3	13	12	13	17	24	11	11	13	15	10	13	South	Shiite	Muslim
89	E3b3	13	12	13	18	24	10	11	13	14	10	13	South	Shiite	Muslim
90	E3b3	14	12	13	19	24	10	11	13	14	10	13	Bekaa	Shiite	Muslim
91	E3b3	13	12	13	17	23	10	11	13	14	10	12	North	Sunnite	Muslim
92	E3b3	13	12	14	18	23	10	11	13	14	10	11	South	Sunnite	Muslim
93	E3b3	13	12	14	19	25	10	11	13	14	10	12	North	Sunnite	Muslim
94	E3b3	13	12	13	18	25	10	11	13	14	10	12	South	Sunnite	Muslim
95	E3b3	13	12	14	17	24	10	11	13	14	10	11	Mt Leb	Sunnite	Muslim
96	E3b3	13	12	12	18	24	10	11	13	14	10	12	North	Sunnite	Muslim
97	E3b3	15	12	13	17	22	10	11	14	14	10	12	North	Sunnite	Muslim
98	G	15	13	12	17	21	10	11	14	15	10	11	Mt Leb	Catholic	Christian
99	G	15	12	12	16	22	10	12	14	16	10	11	Mt Leb	Druze	Druze
100	G	16	12	14	17	23	10	11	13	16	10	12	North	Maronite	Christian
101	G	16	12	12	17	22	9	10	14	16	10	12	North	Maronite	Christian
102	G	15	11	12	19	23	11	12	13	17	10	13	North	Maronite	Christian
103	G	16	12	12	17	22	10	10	14	16	10	11	Mt Leb	Maronite	Christian
104	G	14	12	12	16	24	10	12	13	16	11	11	North	Maronite	Christian
105	G	15	10	12	16	23	10	11	14	16	11	11	North	Maronite	Christian
106	G	15	12	13	16	23	10	11	13	14	10	11	Beirut	Shiite	Muslim
107	G	16	12	12	16	21	10	11	14	16	10	11	Bekaa	Shiite	Muslim
108	G	15	12	14	15	22	11	11	14	16	11	12	South	Shiite	Muslim
109	G	15	12	12	16	21	10	11	13	16	10	11	South	Shiite	Muslim
110	G	15	12	13	18	22	10	11	14	16	10	12	South	Shiite	Muslim
111	G	14	12	12	18	24	12	12	13	16	10	11	South	Shiite	Muslim
112	G	14	12	12	18	24	12	12	13	16	10	11	Beirut	Shiite	Muslim
113	G	15	12	12	17	23	10	11	14	16	10	12	South	Shiite	Muslim
114	G	15	12	12	17	21	11	11	15	16	10	13	South	Shiite	Muslim
115	G	15	12	14	15	22	11	11	14	16	11	12	South	Shiite	Muslim
116	G	14	12	13	16	24	10	12	14	15	10	12	South	Shiite	Muslim
117	G	15	12	12	16	22	10	11	14	16	10	11	South	Shiite	Muslim
118	G	15	12	12	16	22	10	12	13	15	10	11	South	Shiite	Muslim
119	G	15	12	12	17	23	10	11	13	16	10	12	Beirut	Sunnite	Muslim
120	G	16	12	12	16	21	10	10	14	16	10	11	North	Sunnite	Muslim
121	G	15	12	12	16	21	10	11	15	16	10	11	Bekaa	Sunnite	Muslim
122	G	16	12	12	18	23	10	11	14	16	12	11	North	Sunnite	Muslim
123	G	15	12	12	16	21	11	11	14	15	10	13	Beirut	Sunnite	Muslim
124	G	15	12	13	17	22	10	11	13	16	10	12	South	Sunnite	Muslim

125	G	16	12	12	17	22	10	10	14	16	10	11	North	Sunnite	Muslim
126	G	14	12	13	16	23	10	12	12	16	10	11	Mt Leb	Sunnite	Muslim
127	G	15	12	12	17	23	10	11	13	16	10	11	Beirut	Sunnite	Muslim
128	G	15	12	13	16	21	10	11	15	16	11	12	Beirut	Sunnite	Muslim
129	G	16	12	12	17	22	11	10	14	16	10	12	Beirut	Sunnite	Muslim
130	G	15	12	12	17	22	11	11	13	16	10	11	Beirut	Sunnite	Muslim
131	G	16	12	12	17	23	10	12	14	15	10	11	North	Sunnite	Muslim
132	G	14	12	12	17	21	11	11	14	16	10	12	Beirut	Sunnite	Muslim
133	G	16	12	14	18	21	10	11	15	16	10	11	North	Sunnite	Muslim
134	G	14	12	13	16	24	10	12	14	16	10	12	North	Sunnite	Muslim
135	G	16	12	12	17	23	10	11	14	16	10	12	South	Sunnite	Muslim
136	I*(xI1a2,I1a3,I1b2,I1/-c)	15	15	14	17	21	10	11	14	15	11	13	South	Catholic	Christian
137	I*(xI1a2,I1a3,I1b2,I1/-c)	15	12	13	16	22	10	11	12	14	9	12	Mt Leb	Druze	Druze
138	I*(xI1a2,I1a3,I1b2,I1/-c)	15	14	14	16	23	10	13	14	14	10	10	Mt Leb	Druze	Druze
139	I*(xI1a2,I1a3,I1b2,I1/-c)	15	13	13	18	24	11	11	13	15	10	14	North	Maronite	Christian
140	I*(xI1a2,I1a3,I1b2,I1/-c)	15	13	13	18	24	11	11	13	15	10	13	North	Maronite	Christian
141	I*(xI1a2,I1a3,I1b2,I1/-c)	15	13	14	19	23	10	12	14	14	10	11	North	Maronite	Christian
142	I*(xI1a2,I1a3,I1b2,I1/-c)	15	13	13	18	24	11	11	13	15	10	13	North	Maronite	Christian
143	I*(xI1a2,I1a3,I1b2,I1/-c)	14	14	12	16	23	10	11	13	16	10	11	North	Maronite	Christian
144	I*(xI1a2,I1a3,I1b2,I1/-c)	15	13	13	18	24	11	11	13	15	10	12	Beirut	Maronite	Christian
145	I*(xI1a2,I1a3,I1b2,I1/-c)	15	13	13	18	24	11	11	13	15	10	13	North	Maronite	Christian
146	I*(xI1a2,I1a3,I1b2,I1/-c)	14	14	11	17	23	10	11	13	16	10	11	North	Maronite	Christian
147	I*(xI1a2,I1a3,I1b2,I1/-c)	14	14	12	16	23	10	11	13	16	10	11	North	Maronite	Christian
148	I*(xI1a2,I1a3,I1b2,I1/-c)	14	15	14	16	23	10	11	12	14	10	12	Mt Leb	Maronite	Christian
149	I*(xI1a2,I1a3,I1b2,I1/-c)	14	12	13	17	23	10	14	13	14	11	12	North	Maronite	Christian
150	I*(xI1a2,I1a3,I1b2,I1/-c)	15	13	13	17	24	11	11	13	15	10	13	North	Maronite	Christian
151	I*(xI1a2,I1a3,I1b2,I1/-c)	15	13	13	18	24	11	11	13	15	10	13	North	Maronite	Christian
152	I*(xI1a2,I1a3,I1b2,I1/-c)	16	13	14	18	23	10	12	14	14	11	11	North	Orthodox	Christian
153	I*(xI1a2,I1a3,I1b2,I1/-c)	15	17	13	17	23	11	11	12	14	10	11	South	Shiite	Muslim
154	I*(xI1a2,I1a3,I1b2,I1/-c)	13	12	13	18	24	10	11	13	14	10	12	South	Shiite	Muslim
155	I*(xI1a2,I1a3,I1b2,I1/-c)	15	12	13	16	23	10	11	13	14	10	11	Beirut	Sunnite	Muslim
156	I*(xI1a2,I1a3,I1b2,I1/-c)	13	12	13	19	25	10	11	13	14	10	12	North	Sunnite	Muslim
157	I*(xI1a2,I1a3,I1b2,I1/-c)	14	14	12	17	23	10	11	12	14	10	11	Mt Leb	Sunnite	Muslim
158	I*(xI1a2,I1a3,I1b2,I1/-c)	14	12	12	17	22	10	11	14	16	10	11	South	Sunnite	Muslim
159	I*(xI1a2,I1a3,I1b2,I1/-c)	15	13	13	17	23	10	12	15	14	10	12	North	Sunnite	Muslim
160	I*(xI1a2,I1a3,I1b2,I1/-c)	15	12	14	15	23	11	11	14	14	10	12	Beirut	Sunnite	Muslim
161	I*(xI1a2,I1a3,I1b2,I1/-c)	15	12	13	16	23	10	11	13	14	10	11	Bekaa	Sunnite	Muslim
162	J*(xJ2)	15	18	13	18	24	11	11	12	14	10	11	Bekaa	Druze	Druze
163	J*(xJ2)	15	17	13	17	23	11	11	12	14	10	11	Mt Leb	Druze	Druze
164	J*(xJ2)	14	17	13	16	23	10	11	12	14	10	10	North	Maronite	Christian
165	J*(xJ2)	15	16	13	16	23	10	11	12	14	10	11	North	Maronite	Christian
166	J*(xJ2)	16	15	12	16	24	11	11	12	16	9	12	North	Maronite	Christian
167	J*(xJ2)	15	17	13	18	23	11	11	12	14	10	11	North	Maronite	Christian
168	J*(xJ2)	15	16	13	15	24	11	11	12	14	10	12	North	Maronite	Christian
169	J*(xJ2)	14	15	13	16	22	10	11	13	14	9	11	North	Maronite	Christian
170	J*(xJ2)	14	16	13	18	23	10	11	12	14	10	11	North	Maronite	Christian
171	J*(xJ2)	12	16	13	17	23	10	11	12	14	10	12	North	Maronite	Christian
172	J*(xJ2)	14	17	13	17	23	10	10	12	14	10	12	Bekaa	Maronite	Christian
173	J*(xJ2)	15	16	13	17	23	10	11	12	14	10	11	Mt Leb	Maronite	Christian
174	J*(xJ2)	14	15	13	16	22	10	11	12	15	9	11	Mt Leb	Maronite	Christian
175	J*(xJ2)	15	16	13	17	23	10	11	12	14	10	11	North	Maronite	Christian
176	J*(xJ2)	14	16	13	16	23	10	11	12	14	10	11	North	Maronite	Christian
177	J*(xJ2)	14	16	13	17	23	10	11	12	14	10	12	North	Maronite	Christian

178	J*(xJ2)	14	16	13	18	23	10	11	12	14	10	11	Mt Leb	Maronite	Christian
179	J*(xJ2)	14	16	13	16	24	11	11	12	14	10	13	North	Maronite	Christian
180	J*(xJ2)	14	16	13	17	24	10	11	12	14	10	12	Beirut	Maronite	Christian
181	J*(xJ2)	14	16	13	16	24	10	11	12	14	10	12	South	Maronite	Christian
182	J*(xJ2)	15	14	12	16	23	10	11	13	16	10	11	Mt Leb	Maronite	Christian
183	J*(xJ2)	14	13	14	18	23	10	11	12	14	10	12	Mt Leb	Maronite	Christian
184	J*(xJ2)	14	16	14	16	23	10	11	12	14	10	11	North	Maronite	Christian
185	J*(xJ2)	15	16	13	16	23	10	11	12	14	10	11	Mt Leb	Maronite	Christian
186	J*(xJ2)	15	16	13	17	23	10	11	12	14	10	11	North	Maronite	Christian
187	J*(xJ2)	15	17	12	16	23	10	11	13	14	10	11	North	Maronite	Christian
188	J*(xJ2)	15	16	15	17	23	10	11	12	14	10	11	Mt Leb	Maronite	Christian
189	J*(xJ2)	14	16	14	16	24	11	11	12	14	10	12	South	Maronite	Christian
190	J*(xJ2)	14	17	12	17	24	10	11	12	14	10	11	North	Maronite	Christian
191	J*(xJ2)	14	15	14	17	23	10	11	12	14	10	12	North	Maronite	Christian
192	J*(xJ2)	15	16	13	17	23	10	11	12	14	10	11	North	Maronite	Christian
193	J*(xJ2)	14	16	14	16	24	11	11	12	14	10	12	Bekaa	Maronite	Christian
194	J*(xJ2)	13	17	13	17	23	11	11	12	14	10	11	Bekaa	Orthodox	Christian
195	J*(xJ2)	14	15	15	18	23	10	11	12	14	11	12	South	Orthodox	Christian
196	J*(xJ2)	14	15	13	17	23	11	11	12	14	10	12	Mt Leb	Orthodox	Christian
197	J*(xJ2)	14	16	13	18	23	10	11	13	14	10	11	North	Orthodox	Christian
198	J*(xJ2)	15	15	13	17	23	9	11	12	15	7	11	North	Orthodox	Christian
199	J*(xJ2)	13	15	12	17	24	10	11	12	14	10	12	Mt Leb	Shiite	Muslim
200	J*(xJ2)	14	17	13	17	23	11	12	12	14	10	12	Beirut	Shiite	Muslim
201	J*(xJ2)	15	16	13	17	23	11	11	12	14	10	11	South	Shiite	Muslim
202	J*(xJ2)	14	17	13	17	23	10	11	12	14	10	11	Bekaa	Shiite	Muslim
203	J*(xJ2)	14	16	13	17	23	11	11	12	14	10	11	Bekaa	Shiite	Muslim
204	J*(xJ2)	15	17	13	17	23	11	11	12	14	10	11	South	Shiite	Muslim
205	J*(xJ2)	14	12	13	17	23	11	11	12	14	10	11	Mt Leb	Shiite	Muslim
206	J*(xJ2)	14	13	13	16	23	10	11	13	14	10	13	South	Shiite	Muslim
207	J*(xJ2)	14	16	13	17	23	10	11	12	14	10	13	South	Shiite	Muslim
208	J*(xJ2)	14	17	14	17	23	11	11	12	14	10	11	South	Shiite	Muslim
209	J*(xJ2)	15	17	13	17	23	11	11	12	14	10	11	South	Shiite	Muslim
210	J*(xJ2)	14	15	13	17	22	10	11	13	15	9	10	South	Shiite	Muslim
211	J*(xJ2)	14	16	15	18	23	10	11	12	14	10	11	South	Shiite	Muslim
212	J*(xJ2)	14	16	13	17	23	10	11	12	14	10	12	South	Shiite	Muslim
213	J*(xJ2)	14	17	13	16	23	12	11	12	14	10	12	South	Shiite	Muslim
214	J*(xJ2)	15	17	13	18	23	10	11	12	14	10	11	South	Shiite	Muslim
215	J*(xJ2)	14	16	14	17	23	10	11	12	14	10	11	Bekaa	Shiite	Muslim
216	J*(xJ2)	14	17	13	17	22	10	11	12	14	10	11	South	Shiite	Muslim
217	J*(xJ2)	14	14	13	16	24	10	11	12	14	10	12	South	Shiite	Muslim
218	J*(xJ2)	14	16	13	17	23	10	11	12	14	10	11	South	Shiite	Muslim
219	J*(xJ2)	14	16	13	17	23	11	11	12	14	10	12	South	Shiite	Muslim
220	J*(xJ2)	15	15	13	17	23	10	11	12	14	10	11	Bekaa	Shiite	Muslim
221	J*(xJ2)	15	17	13	18	23	11	11	12	14	10	11	Bekaa	Shiite	Muslim
222	J*(xJ2)	14	16	13	17	22	10	11	12	14	10	11	South	Shiite	Muslim
223	J*(xJ2)	15	17	13	18	23	11	11	12	14	10	11	South	Shiite	Muslim
224	J*(xJ2)	14	17	12	16	23	10	11	12	16	9	11	Bekaa	Shiite	Muslim
225	J*(xJ2)	14	15	13	17	22	10	11	13	15	9	10	South	Shiite	Muslim
226	J*(xJ2)	14	17	14	15	23	11	11	12	14	10	11	South	Shiite	Muslim
227	J*(xJ2)	15	16	13	16	23	9	11	12	14	9	12	South	Shiite	Muslim
228	J*(xJ2)	14	13	14	16	23	10	11	12	14	10	11	South	Shiite	Muslim
229	J*(xJ2)	14	17	13	16	23	11	11	12	14	10	12	South	Shiite	Muslim
230	J*(xJ2)	14	16	14	18	24	10	11	12	14	10	11	Bekaa	Shiite	Muslim

231	J*(xJ2)	14	17	13	16	22	11	11	12	14	10	11	South	Shiite	Muslim
232	J*(xJ2)	14	17	13	17	23	11	11	12	14	10	13	Bekaa	Shiite	Muslim
233	J*(xJ2)	14	17	13	16	23	11	11	12	14	10	12	South	Shiite	Muslim
234	J*(xJ2)	14	16	13	16	23	10	11	12	14	10	11	Beirut	Shiite	Muslim
235	J*(xJ2)	15	17	14	18	23	11	11	12	14	11	11	South	Shiite	Muslim
236	J*(xJ2)	15	17	14	17	24	11	11	12	14	10	11	South	Shiite	Muslim
237	J*(xJ2)	14	17	14	16	23	10	11	12	14	10	12	South	Shiite	Muslim
238	J*(xJ2)	15	17	13	18	23	12	11	12	14	10	11	South	Shiite	Muslim
239	J*(xJ2)	14	12	13	16	23	10	11	12	15	9	12	North	Sunnite	Muslim
240	J*(xJ2)	14	15	13	16	23	10	11	12	15	9	12	Beirut	Sunnite	Muslim
241	J*(xJ2)	14	13	13	16	23	10	11	12	14	10	12	Beirut	Sunnite	Muslim
242	J*(xJ2)	14	17	14	17	23	11	11	12	14	10	11	North	Sunnite	Muslim
243	J*(xJ2)	14	17	13	17	23	11	11	12	14	10	11	North	Sunnite	Muslim
244	J*(xJ2)	14	17	14	17	24	10	11	12	14	10	11	North	Sunnite	Muslim
245	J*(xJ2)	14	17	13	17	23	10	11	12	14	10	12	North	Sunnite	Muslim
246	J*(xJ2)	14	15	13	16	23	10	11	12	15	9	11	South	Sunnite	Muslim
247	J*(xJ2)	14	17	13	16	23	11	11	12	14	10	11	North	Sunnite	Muslim
248	J*(xJ2)	14	17	14	17	23	11	11	12	14	10	11	North	Sunnite	Muslim
249	J*(xJ2)	14	17	12	16	22	11	11	12	14	10	11	Beirut	Sunnite	Muslim
250	J*(xJ2)	14	16	13	15	23	10	11	12	14	10	11	Bekaa	Sunnite	Muslim
251	J*(xJ2)	14	17	13	16	22	11	11	12	14	10	11	North	Sunnite	Muslim
252	J*(xJ2)	14	15	13	16	23	11	11	12	15	9	11	Beirut	Sunnite	Muslim
253	J*(xJ2)	14	17	13	17	23	10	12	12	14	10	11	South	Sunnite	Muslim
254	J*(xJ2)	15	17	13	17	23	11	11	12	14	11	11	Beirut	Sunnite	Muslim
255	J*(xJ2)	14	15	14	16	24	10	11	12	14	10	12	South	Sunnite	Muslim
256	J*(xJ2)	14	16	13	17	23	10	11	13	14	10	11	North	Sunnite	Muslim
257	J*(xJ2)	14	16	13	17	23	11	11	12	14	10	11	Beirut	Sunnite	Muslim
258	J*(xJ2)	14	17	13	17	23	10	11	12	14	9	11	Beirut	Sunnite	Muslim
259	J*(xJ2)	14	17	13	17	23	11	11	12	14	10	11	Mt Leb	Sunnite	Muslim
260	J*(xJ2)	15	17	13	17	23	11	11	12	14	10	12	North	Sunnite	Muslim
261	J*(xJ2)	14	16	13	16	23	11	11	12	14	10	11	North	Sunnite	Muslim
262	J*(xJ2)	14	15	13	18	23	11	11	12	15	9	14	Bekaa	Sunnite	Muslim
263	J*(xJ2)	14	15	14	16	25	10	11	12	16	10	11	North	Sunnite	Muslim
264	J*(xJ2)	14	15	13	16	23	11	11	12	14	10	11	Beirut	Sunnite	Muslim
265	J*(xJ2)	14	17	13	16	23	11	11	12	14	10	11	North	Sunnite	Muslim
266	J*(xJ2)	14	16	13	16	23	10	11	13	14	10	12	North	Sunnite	Muslim
267	J*(xJ2)	14	17	13	17	23	11	11	12	14	10	11	Bekaa	Sunnite	Muslim
268	J*(xJ2)	14	17	13	16	23	11	11	12	14	10	11	Bekaa	Sunnite	Muslim
269	J*(xJ2)	14	17	13	16	22	11	11	11	14	10	11	Beirut	Sunnite	Muslim
270	J*(xJ2)	14	17	13	17	23	11	11	12	14	10	11	South	Sunnite	Muslim
271	J*(xJ2)	14	13	14	15	23	10	11	12	14	10	11	North	Sunnite	Muslim
272	J*(xJ2)	14	17	14	17	23	11	11	12	14	10	12	North	Sunnite	Muslim
273	J*(xJ2)	15	16	13	17	23	10	11	12	14	10	11	Beirut	Sunnite	Muslim
274	J*(xJ2)	14	17	13	16	22	11	11	11	14	10	11	North	Sunnite	Muslim
275	J*(xJ2)	14	14	12	17	23	10	11	12	14	10	11	Mt Leb	Sunnite	Muslim
276	J*(xJ2)	14	15	13	17	23	10	11	12	14	10	11	Beirut	Sunnite	Muslim
277	J*(xJ2)	14	17	13	17	23	11	11	12	14	10	12	Beirut	Sunnite	Muslim
278	J*(xJ2)	14	15	14	16	25	10	11	12	16	10	11	North	Sunnite	Muslim
279	J*(xJ2)	14	15	14	17	23	10	11	12	15	9	11	North	Sunnite	Muslim
280	J*(xJ2)	14	15	12	17	23	11	11	12	15	10	12	South	Sunnite	Muslim
281	J*(xJ2)	14	16	13	16	23	10	11	12	14	10	11	Beirut	Sunnite	Muslim
282	J2/-f1*(xJ2f1a)	14	15	13	18	23	10	11	12	15	9	12	Beirut	Assyrian Orth.	Christian
283	J2/-f1*(xJ2f1a)	14	15	13	17	23	10	11	12	15	9	11	Bekaa	Catholic	Christian

284	J2/-f1*(xJ2f1a)	15	15	13	16	22	10	11	12	15	9	11	South	Catholic	Christian
285	J2/-f1*(xJ2f1a)	15	15	13	17	23	9	11	12	15	7	11	South	Catholic	Christian
286	J2/-f1*(xJ2f1a)	14	16	13	16	24	10	11	12	15	9	11	South	Catholic	Christian
287	J2/-f1*(xJ2f1a)	14	15	13	16	22	10	11	12	15	9	11	North	Maronite	Christian
288	J2/-f1*(xJ2f1a)	14	15	13	16	23	10	11	12	15	9	10	North	Maronite	Christian
289	J2/-f1*(xJ2f1a)	14	15	13	16	23	10	11	12	15	9	10	North	Maronite	Christian
290	J2/-f1*(xJ2f1a)	15	16	14	15	23	11	12	12	15	9	11	North	Maronite	Christian
291	J2/-f1*(xJ2f1a)	14	15	13	17	23	10	11	12	15	9	10	North	Maronite	Christian
292	J2/-f1*(xJ2f1a)	14	15	13	16	23	10	11	12	15	9	10	North	Maronite	Christian
293	J2/-f1*(xJ2f1a)	14	15	13	16	22	10	11	12	15	9	11	North	Maronite	Christian
294	J2/-f1*(xJ2f1a)	14	15	13	16	23	9	11	12	15	9	11	North	Maronite	Christian
295	J2/-f1*(xJ2f1a)	14	15	13	16	23	10	11	12	15	9	10	North	Maronite	Christian
296	J2/-f1*(xJ2f1a)	14	16	13	16	23	10	11	12	15	9	11	North	Maronite	Christian
297	J2/-f1*(xJ2f1a)	15	15	13	16	22	10	11	12	15	9	11	Mt Leb	Maronite	Christian
298	J2/-f1*(xJ2f1a)	14	15	13	16	22	10	11	12	15	9	11	Mt Leb	Maronite	Christian
299	J2/-f1*(xJ2f1a)	14	15	13	16	22	10	11	13	14	9	11	South	Maronite	Christian
300	J2/-f1*(xJ2f1a)	14	15	12	16	22	10	11	12	15	9	11	North	Maronite	Christian
301	J2/-f1*(xJ2f1a)	14	15	13	16	22	10	11	12	15	9	11	North	Maronite	Christian
302	J2/-f1*(xJ2f1a)	14	15	13	16	23	10	11	12	15	9	10	North	Maronite	Christian
303	J2/-f1*(xJ2f1a)	15	15	13	17	23	9	11	12	15	7	11	North	Maronite	Christian
304	J2/-f1*(xJ2f1a)	14	15	13	16	23	10	11	12	15	9	11	North	Maronite	Christian
305	J2/-f1*(xJ2f1a)	15	15	13	17	23	9	11	12	15	7	11	North	Maronite	Christian
306	J2/-f1*(xJ2f1a)	15	15	14	16	22	10	11	12	15	9	11	Bekaa	Maronite	Christian
307	J2/-f1*(xJ2f1a)	14	15	13	16	23	9	11	12	15	9	11	North	Maronite	Christian
308	J2/-f1*(xJ2f1a)	15	15	13	16	22	10	11	12	15	9	11	Mt Leb	Maronite	Christian
309	J2/-f1*(xJ2f1a)	14	15	13	16	22	11	11	12	15	9	13	North	Maronite	Christian
310	J2/-f1*(xJ2f1a)	14	15	13	16	22	10	11	12	15	9	11	Mt Leb	Maronite	Christian
311	J2/-f1*(xJ2f1a)	14	15	13	16	23	10	11	12	15	9	11	North	Maronite	Christian
312	J2/-f1*(xJ2f1a)	14	15	13	16	23	10	11	12	15	9	10	North	Maronite	Christian
313	J2/-f1*(xJ2f1a)	14	13	13	16	23	10	11	12	15	9	11	North	Orthodox	Christian
314	J2/-f1*(xJ2f1a)	15	15	13	16	23	10	11	12	15	9	13	Bekaa	Shiite	Muslim
315	J2/-f1*(xJ2f1a)	14	15	13	17	23	9	11	12	14	7	11	South	Shiite	Muslim
316	J2/-f1*(xJ2f1a)	14	15	13	16	23	10	11	12	14	9	12	South	Shiite	Muslim
317	J2/-f1*(xJ2f1a)	14	15	13	17	22	10	11	12	15	9	11	Bekaa	Shiite	Muslim
318	J2/-f1*(xJ2f1a)	14	15	13	16	23	10	11	12	15	9	11	South	Shiite	Muslim
319	J2/-f1*(xJ2f1a)	14	15	13	18	22	10	11	12	15	9	12	Bekaa	Shiite	Muslim
320	J2/-f1*(xJ2f1a)	15	15	13	16	25	10	11	12	15	9	11	South	Sunnite	Muslim
321	J2/-f1*(xJ2f1a)	14	15	13	18	24	10	11	12	15	9	13	North	Sunnite	Muslim
322	J2/-f1*(xJ2f1a)	14	15	12	17	22	11	11	12	14	9	11	North	Sunnite	Muslim
323	J2/-f1*(xJ2f1a)	14	15	13	17	22	10	11	12	14	9	11	Mt Leb	Sunnite	Muslim
324	J2/-f1*(xJ2f1a)	15	15	13	17	23	10	12	12	14	10	11	Mt Leb	Sunnite	Muslim
325	J2/-f1*(xJ2f1a)	15	16	13	17	23	10	11	12	14	9	11	Beirut	Sunnite	Muslim
326	J2/-f1*(xJ2f1a)	14	15	13	16	22	10	11	12	14	9	11	North	Sunnite	Muslim
327	J2/-f1a	15	15	14	17	24	10	11	12	15	9	14	Bekaa	Shiite	Muslim
328	J2a	14	12	13	17	22	10	11	14	16	10	11	Bekaa	Catholic	Christian
329	J2a	14	15	14	15	22	9	11	12	15	9	12	South	Catholic	Christian
330	J2a	13	15	13	16	23	10	11	12	14	9	11	Mt Leb	Druze	Druze
331	J2a	14	14	13	17	24	10	11	12	15	10	12	Mt Leb	Druze	Druze
332	J2a	15	16	13	16	23	9	11	12	14	9	13	South	Druze	Druze
333	J2a	14	14	13	17	24	10	11	12	15	9	12	North	Maronite	Christian
334	J2a	14	15	14	16	25	10	11	12	16	10	12	North	Maronite	Christian
335	J2a	14	14	13	17	24	10	11	12	15	9	12	North	Maronite	Christian
336	J2a	14	17	14	15	25	10	11	13	14	9	11	South	Maronite	Christian

337	J2a	15	15	14	18	25	10	11	12	15	9	11	North	Maronite	Christian
338	J2a	16	15	15	18	25	10	11	12	15	9	12	Mt Leb	Maronite	Christian
339	J2a	15	16	13	16	23	9	11	12	14	9	12	North	Maronite	Christian
340	J2a	15	16	13	16	23	9	11	12	14	9	12	North	Maronite	Christian
341	J2a	14	14	13	17	24	10	11	12	15	9	12	North	Maronite	Christian
342	J2a	14	17	12	17	23	10	11	12	15	9	11	Mt Leb	Maronite	Christian
343	J2a	15	15	14	18	25	10	11	12	15	9	11	Mt Leb	Maronite	Christian
344	J2a	14	15	14	15	22	9	11	12	15	9	11	Bekaa	Maronite	Christian
345	J2a	15	15	13	16	22	10	11	12	15	9	11	Mt Leb	Maronite	Christian
346	J2a	15	16	13	16	23	9	11	12	14	9	12	North	Maronite	Christian
347	J2a	15	15	13	16	24	10	11	12	15	9	13	Mt Leb	Maronite	Christian
348	J2a	14	15	13	16	25	10	11	12	16	10	11	North	Maronite	Christian
349	J2a	15	14	13	16	23	9	11	12	14	9	13	North	Maronite	Christian
350	J2a	14	13	14	16	24	10	11	12	14	9	12	North	Maronite	Christian
351	J2a	14	15	13	16	23	10	11	12	15	9	11	North	Maronite	Christian
352	J2a	14	13	14	15	22	9	11	12	14	9	11	North	Maronite	Christian
353	J2a	15	16	13	16	23	9	11	12	14	9	13	North	Maronite	Christian
354	J2a	15	16	13	16	23	9	11	12	14	9	12	North	Maronite	Christian
355	J2a	14	13	13	16	24	10	11	12	14	9	12	Mt Leb	Maronite	Christian
356	J2a	15	16	13	17	23	8	11	12	14	9	12	Mt Leb	Maronite	Christian
357	J2a	14	16	13	18	23	10	11	12	14	10	11	Mt Leb	Maronite	Christian
358	J2a	14	14	13	17	24	10	11	12	15	9	12	North	Orthodox	Christian
359	J2a	15	16	13	16	23	9	11	12	14	9	12	North	Orthodox	Christian
360	J2a	15	16	13	16	23	9	11	12	14	9	12	North	Orthodox	Christian
361	J2a	14	14	12	16	23	10	11	12	15	9	13	North	Orthodox	Christian
362	J2a	14	13	13	20	22	11	11	14	15	9	12	Bekaa	Shiite	Muslim
363	J2a	14	15	13	15	24	11	11	12	15	9	11	Mt Leb	Shiite	Muslim
364	J2a	14	16	12	16	24	11	11	12	15	9	12	South	Shiite	Muslim
365	J2a	14	16	12	16	23	10	11	12	16	9	11	Bekaa	Shiite	Muslim
366	J2a	14	15	13	16	23	10	11	12	15	9	11	South	Shiite	Muslim
367	J2a	15	15	13	16	25	11	11	12	15	9	12	South	Shiite	Muslim
368	J2a	15	14	13	16	24	10	11	12	15	9	12	South	Shiite	Muslim
369	J2a	14	15	13	16	23	10	11	12	15	9	11	South	Shiite	Muslim
370	J2a	14	15	13	16	23	10	11	12	15	9	11	South	Shiite	Muslim
371	J2a	14	15	13	16	23	11	11	12	15	9	11	South	Shiite	Muslim
372	J2a	15	16	13	16	23	9	11	12	14	9	12	South	Shiite	Muslim
373	J2a	15	16	13	16	23	9	11	12	14	9	12	South	Shiite	Muslim
374	J2a	14	14	13	17	23	10	11	12	15	9	11	South	Shiite	Muslim
375	J2a	15	15	13	16	24	11	11	12	15	9	12	South	Shiite	Muslim
376	J2a	14	15	12	17	25	10	11	15	15	9	12	South	Shiite	Muslim
377	J2a	15	12	13	18	21	10	11	15	14	12	12	South	Shiite	Muslim
378	J2a	14	17	12	16	23	10	11	12	16	9	11	Bekaa	Shiite	Muslim
379	J2a	14	17	12	16	23	10	11	12	16	9	11	Beirut	Shiite	Muslim
380	J2a	14	14	13	17	24	10	11	12	14	9	12	North	Shiite	Muslim
381	J2a	14	15	12	16	23	10	11	12	16	9	11	Bekaa	Shiite	Muslim
382	J2a	14	16	12	16	24	11	11	12	14	9	12	South	Sunnite	Muslim
383	J2a	14	16	12	16	24	11	11	12	14	9	12	South	Sunnite	Muslim
384	J2a	15	14	13	16	23	10	11	12	15	9	11	South	Sunnite	Muslim
385	J2a	14	15	13	16	23	10	11	12	15	9	11	Mt Leb	Sunnite	Muslim
386	J2a	16	16	14	17	23	9	11	12	14	9	12	North	Sunnite	Muslim
387	J2a	14	15	13	16	23	10	11	12	15	9	12	Bekaa	Sunnite	Muslim
388	J2a	14	15	13	16	23	10	11	12	15	9	12	North	Sunnite	Muslim
389	J2a	14	14	13	17	23	10	11	12	15	9	13	North	Sunnite	Muslim

390	J2a	14	15	12	16	23	10	11	13	15	10	12	Beirut	Sunnite	Muslim
391	J2a	15	15	13	17	23	10	11	13	15	10	12	Bekaa	Sunnite	Muslim
392	J2a	14	14	14	16	23	10	11	12	14	9	12	South	Sunnite	Muslim
393	J2a	14	15	13	16	23	10	11	12	16	9	11	Mt Leb	Sunnite	Muslim
394	J2a	16	16	13	16	23	9	11	12	14	9	12	North	Sunnite	Muslim
395	J2a	14	14	13	17	23	10	11	12	16	9	11	Beirut	Sunnite	Muslim
396	J2a	15	12	13	16	22	10	11	12	14	9	11	North	Sunnite	Muslim
397	J2a	14	13	12	17	23	10	11	12	15	9	11	North	Sunnite	Muslim
398	J2a	14	15	14	14	22	9	11	12	15	9	11	North	Sunnite	Muslim
399	J2a	14	16	13	16	22	10	11	12	15	9	11	Mt Leb	Sunnite	Muslim
400	J2a	14	15	13	17	23	10	11	12	15	9	12	South	Sunnite	Muslim
401	J2a	14	15	13	16	23	10	11	12	15	9	12	North	Sunnite	Muslim
402	J2a	14	15	13	16	23	10	11	12	15	9	12	North	Sunnite	Muslim
403	J2a	14	18	12	16	23	11	11	12	15	9	12	Beirut	Sunnite	Muslim
404	J2a	14	14	12	17	24	11	11	12	15	9	13	Bekaa	Sunnite	Muslim
405	J2a	14	16	13	18	23	11	11	12	15	9	14	North	Sunnite	Muslim
406	J2a	14	18	13	16	23	10	11	12	14	9	9	Beirut	Sunnite	Muslim
407	J2a	14	15	12	16	23	10	11	13	15	10	12	Beirut	Sunnite	Muslim
408	J2a	14	15	13	16	23	11	11	12	15	9	11	South	Sunnite	Muslim
409	J2a	14	14	14	16	25	10	11	12	15	9	12	Beirut	Sunnite	Muslim
410	J2a	14	16	14	16	22	9	11	12	15	9	11	North	Sunnite	Muslim
411	J2a	14	15	13	16	23	10	11	12	15	9	11	North	Sunnite	Muslim
412	J2a	14	17	12	17	23	11	11	12	15	9	12	North	Sunnite	Muslim
413	J2a	14	16	13	16	23	10	11	12	15	9	12	South	Sunnite	Muslim
414	J2e*(xJ2e1)	17	15	12	16	24	11	11	12	16	9	12	Mt Leb	Maronite	Christian
415	J2e*(xJ2e1)	16	15	12	16	24	10	11	12	16	9	12	Mt Leb	Maronite	Christian
416	J2e*(xJ2e1)	16	14	12	16	24	11	11	12	16	9	12	South	Maronite	Christian
417	J2e*(xJ2e1)	16	15	13	16	24	11	11	12	16	9	13	Bekaa	Maronite	Christian
418	J2e*(xJ2e1)	16	15	12	16	25	11	11	12	16	9	12	North	Maronite	Christian
419	J2e*(xJ2e1)	15	15	11	16	24	11	11	12	16	9	12	Mt Leb	Maronite	Christian
420	J2e*(xJ2e1)	16	15	12	16	24	11	11	12	16	9	12	North	Maronite	Christian
421	J2e*(xJ2e1)	14	15	14	15	22	9	11	12	15	9	11	North	Maronite	Christian
422	J2e*(xJ2e1)	16	15	12	16	24	11	11	12	16	9	12	North	Maronite	Christian
423	J2e*(xJ2e1)	16	16	12	17	24	10	11	12	16	9	11	North	Maronite	Christian
424	J2e*(xJ2e1)	16	15	12	16	23	10	11	12	16	9	12	North	Maronite	Christian
425	J2e*(xJ2e1)	16	15	12	16	23	10	11	12	16	9	12	North	Maronite	Christian
426	J2e*(xJ2e1)	16	15	12	16	24	11	11	12	16	9	12	South	Maronite	Christian
427	J2e*(xJ2e1)	16	15	12	16	24	11	11	12	16	9	12	Bekaa	Shiite	Muslim
428	J2e*(xJ2e1)	15	15	12	16	24	10	11	12	14	9	12	South	Shiite	Muslim
429	J2e*(xJ2e1)	15	15	12	17	25	10	12	12	15	9	11	Beirut	Sunnite	Muslim
430	J2e*(xJ2e1)	16	15	12	15	24	10	11	12	16	9	11	South	Sunnite	Muslim
431	K*(xK2,K3,L,M,NO,P)	15	12	14	15	24	10	13	13	15	8	12	Mt Leb	Maronite	Christian
432	K*(xK2,K3,L,M,NO,P)	13	12	12	19	24	10	11	13	14	10	12	South	Shiite	Muslim
433	K*(xK2,K3,L,M,NO,P)	14	15	13	16	23	10	11	12	15	9	11	South	Sunnite	Muslim
434	K*(xK2,K3,L,M,NO,P)	14	12	12	16	22	10	14	11	15	10	13	North	Sunnite	Muslim
435	K2	14	12	13	16	23	10	13	13	14	9	11	Mt Leb	Arm. Orth.	Christian
436	K2	14	12	13	17	23	10	16	14	15	9	11	Bekaa	Catholic	Christian
437	K2	14	12	13	18	23	10	13	13	14	9	11	North	Catholic	Christian
438	K2	15	12	13	16	23	10	13	13	14	9	11	Mt Leb	Maronite	Christian
439	K2	15	12	14	16	24	10	13	13	14	9	12	North	Maronite	Christian
440	K2	14	12	14	16	23	10	13	13	14	9	11	Mt Leb	Maronite	Christian
441	K2	15	12	13	16	23	10	13	13	14	9	11	Mt Leb	Maronite	Christian
442	K2	15	12	14	16	24	10	13	13	14	9	12	North	Maronite	Christian

443	K2	15	12	14	16	24	10	13	13	14	9	12	North	Maronite	Christian
444	K2	15	12	13	16	23	10	13	13	14	9	11	Bekaa	Maronite	Christian
445	K2	15	12	14	16	24	10	13	13	14	9	12	North	Maronite	Christian
446	K2	15	12	13	16	23	10	13	13	14	9	11	North	Orthodox	Christian
447	K2	14	12	14	16	23	10	13	13	14	9	11	South	Shiite	Muslim
448	K2	14	12	13	17	24	10	13	13	14	9	12	South	Shiite	Muslim
449	K2	15	12	14	16	23	10	13	13	14	9	11	South	Shiite	Muslim
450	K2	13	12	14	14	23	10	13	13	14	9	12	Bekaa	Shiite	Muslim
451	K2	15	12	14	16	23	10	13	13	14	9	11	South	Shiite	Muslim
452	K2	14	12	13	17	23	10	13	13	14	9	11	South	Shiite	Muslim
453	K2	14	12	14	17	23	11	13	13	15	9	12	Beirut	Sunnite	Muslim
454	K2	15	12	13	17	23	10	13	13	14	9	11	North	Sunnite	Muslim
455	K2	14	12	14	15	24	10	13	13	14	9	11	Bekaa	Sunnite	Muslim
456	K2	15	12	14	16	23	10	13	13	14	9	11	Beirut	Sunnite	Muslim
457	K2	15	12	12	15	24	10	14	13	14	9	10	North	Sunnite	Muslim
458	L*	14	12	13	16	24	10	14	12	16	8	10	Mt Leb	Druze	Druze
459	L*	14	12	13	17	23	10	14	12	15	10	12	Mt Leb	Druze	Druze
460	L*	14	12	12	16	23	10	14	11	15	10	13	Mt Leb	Druze	Druze
461	L*	15	12	14	15	25	10	14	12	16	10	11	North	Maronite	Christian
462	L*	14	12	13	16	23	10	14	12	16	10	12	North	Maronite	Christian
463	L*	14	12	13	16	24	10	14	12	16	10	12	North	Maronite	Christian
464	L*	14	12	13	16	23	10	14	12	16	10	12	North	Maronite	Christian
465	L*	14	12	13	17	23	10	14	12	16	10	12	North	Maronite	Christian
466	L*	14	12	13	16	23	10	14	12	16	10	12	North	Maronite	Christian
467	L*	14	12	13	16	23	10	14	12	16	10	12	North	Maronite	Christian
468	L*	14	12	13	16	23	10	14	12	16	10	12	North	Maronite	Christian
469	L*	14	12	13	16	23	10	14	12	16	10	12	North	Maronite	Christian
470	L*	14	12	13	17	22	10	14	12	15	10	12	North	Maronite	Christian
471	L*	14	12	13	16	23	10	14	12	16	10	12	North	Maronite	Christian
472	L*	15	12	13	15	25	10	14	12	16	10	11	Bekaa	Maronite	Christian
473	L*	14	12	12	16	23	10	14	12	16	10	12	North	Maronite	Christian
474	L*	15	12	13	15	25	10	14	11	16	10	11	South	Maronite	Christian
475	L*	14	12	13	16	23	10	14	12	16	10	13	North	Maronite	Christian
476	L*	14	12	13	16	23	10	14	12	16	10	12	North	Maronite	Christian
477	L*	14	12	13	16	23	10	14	12	16	10	12	North	Maronite	Christian
478	L*	14	12	13	17	23	10	14	12	16	10	13	North	Maronite	Christian
479	L*	14	12	13	16	23	10	14	12	16	10	12	North	Maronite	Christian
480	L*	15	12	14	15	23	10	14	11	16	10	8	North	Orthodox	Christian
481	L*	14	12	13	17	22	10	14	11	15	10	10	Bekaa	Shiite	Muslim
482	L*	14	12	13	17	22	10	14	12	15	10	11	Bekaa	Shiite	Muslim
483	L*	14	12	13	17	22	10	14	11	15	10	11	Bekaa	Shiite	Muslim
484	L*	14	12	13	17	22	10	14	11	15	10	11	Bekaa	Shiite	Muslim
485	L*	15	12	14	15	25	10	14	11	16	10	11	North	Shiite	Muslim
486	L*	14	12	13	17	22	10	14	11	15	10	11	Mt Leb	Shiite	Muslim
487	L*	15	12	13	17	23	11	13	11	15	10	12	North	Sunnite	Muslim
488	L*	15	12	13	15	25	10	14	11	16	10	11	North	Sunnite	Muslim
489	L*	15	12	14	15	25	10	14	12	16	10	11	North	Sunnite	Muslim
490	L*	14	12	14	16	23	10	13	12	16	10	12	North	Sunnite	Muslim
491	N or O	14	12	13	16	23	10	14	13	14	10	10	Beirut	Sunnite	Muslim
492	Q*	13	12	14	16	22	10	14	13	14	11	12	Mt Leb	Druze	Druze
493	Q*	14	10	13	17	23	10	14	13	14	11	12	Mt Leb	Maronite	Christian
494	Q*	14	12	13	17	23	10	14	13	14	11	12	Mt Leb	Maronite	Christian
495	Q*	14	12	13	17	25	10	13	13	16	11	12	Bekaa	Shiite	Muslim

496	Q*	14	12	14	17	23	10	14	13	14	11	12	Bekaa	Shiite	Muslim
497	Q*	14	12	14	17	23	10	14	13	14	11	13	Bekaa	Shiite	Muslim
498	Q*	14	12	12	17	25	10	14	13	14	11	11	Mt Leb	Sunnite	Muslim
499	Q*	13	12	13	18	24	10	12	12	13	11	11	North	Sunnite	Muslim
500	Q*	13	12	13	18	24	10	14	13	13	11	12	North	Sunnite	Muslim
501	Q2	15	12	13	16	23	10	16	13	14	11	12	Beirut	Shiite	Muslim
502	Q2	15	12	13	16	24	10	17	13	14	11	12	South	Shiite	Muslim
503	R*	12	12	13	15	24	10	14	12	16	12	12	North	Maronite	Christian
504	R*	14	12	12	16	24	10	13	13	15	12	13	North	Maronite	Christian
505	R*	13	12	13	16	25	11	13	12	15	12	13	North	Maronite	Christian
506	R*	14	12	13	15	24	11	13	12	15	12	11	South	Shiite	Muslim
507	R*	14	12	13	16	25	10	13	12	15	11	11	Mt Leb	Sunnite	Muslim
508	R*	14	12	13	16	24	10	13	12	15	12	12	Mt Leb	Sunnite	Muslim
509	R*	15	12	11	16	24	10	13	13	15	11	11	Beirut	Sunnite	Muslim
510	R*	14	12	14	16	23	10	12	13	14	11	12	North	Sunnite	Muslim
511	R*	15	12	13	18	23	11	13	13	15	11	11	Bekaa	Sunnite	Muslim
512	R*	14	12	13	16	24	10	13	12	15	12	13	Beirut	Sunnite	Muslim
513	R1/-b*	14	12	13	17	23	11	13	13	15	12	11	North	Orthodox	Christian
514	R1/-b1a	15	13	13	16	24	10	13	12	15	11	12	Mt Leb	Druze	Druze
515	R1/-b1a	16	13	13	16	23	10	13	13	15	11	12	South	Shiite	Muslim
516	R1/-b1a	15	11	14	15	24	10	13	14	14	12	13	South	Sunnite	Muslim
517	R1/-b1c	14	12	13	16	24	11	13	12	15	12	12	Mt Leb	Arm. Cath.	Christian
518	R1/-b1c	14	12	13	16	24	10	13	12	15	12	12	Mt Leb	Arm. Orth.	Christian
519	R1/-b1c	14	12	13	16	24	11	14	12	15	12	11	Mt Leb	Arm. Orth.	Christian
520	R1/-b1c	14	12	13	17	23	11	13	12	15	12	12	Beirut	Arm. Orth.	Christian
521	R1/-b1c	14	12	13	18	24	10	12	12	15	12	14	North	Christian	Christian
522	R1/-b1c	14	12	13	16	24	11	13	13	15	12	13	Mt Leb	Druze	Druze
523	R1/-b1c	14	12	13	16	24	11	14	12	15	13	12	Bekaa	Druze	Druze
524	R1/-b1c	14	12	13	15	24	11	14	12	15	13	12	Bekaa	Druze	Druze
525	R1/-b1c	14	12	12	16	24	11	13	12	15	12	12	Beirut	Maronite	Christian
526	R1/-b1c	14	12	12	17	24	11	13	12	15	12	13	North	Maronite	Christian
527	R1/-b1c	14	12	12	16	24	10	13	13	15	12	12	North	Maronite	Christian
528	R1/-b1c	14	12	12	16	24	11	13	12	15	12	14	Mt Leb	Maronite	Christian
529	R1/-b1c	14	12	12	16	24	10	13	13	15	12	13	North	Maronite	Christian
530	R1/-b1c	14	12	13	16	25	10	13	12	15	11	11	Mt Leb	Maronite	Christian
531	R1/-b1c	14	12	12	16	24	11	13	13	15	12	13	North	Maronite	Christian
532	R1/-b1c	12	12	13	15	24	10	14	12	16	12	12	North	Maronite	Christian
533	R1/-b1c	14	12	13	19	24	10	12	12	15	12	13	Mt Leb	Maronite	Christian
534	R1/-b1c	14	12	12	16	24	10	13	13	15	12	13	North	Maronite	Christian
535	R1/-b1c	14	12	12	16	24	10	13	11	15	12	13	North	Maronite	Christian
536	R1/-b1c	11	12	13	15	24	11	14	12	16	12	12	South	Maronite	Christian
537	R1/-b1c	15	12	13	16	25	11	13	15	15	12	11	Mt Leb	Maronite	Christian
538	R1/-b1c	14	12	12	16	24	11	13	13	15	12	13	North	Maronite	Christian
539	R1/-b1c	14	12	14	16	24	10	13	12	15	12	12	North	Maronite	Christian
540	R1/-b1c	14	12	12	16	24	10	13	11	15	12	13	North	Maronite	Christian
541	R1/-b1c	14	12	12	16	24	10	13	13	15	12	13	North	Maronite	Christian
542	R1/-b1c	14	12	12	16	24	10	13	13	15	12	13	North	Maronite	Christian
543	R1/-b1c	14	12	12	16	24	11	13	13	15	12	13	North	Maronite	Christian
544	R1/-b1c	14	12	14	16	25	10	13	12	15	12	12	Mt Leb	Maronite	Christian
545	R1/-b1c	14	12	13	16	25	10	13	12	15	12	12	North	Orthodox	Christian
546	R1/-b1c	14	12	13	17	24	10	13	12	15	12	12	North	Shiite	Muslim
547	R1/-b1c	14	12	13	16	23	10	13	12	15	11	11	Bekaa	Shiite	Muslim
548	R1/-b1c	14	12	13	16	24	11	13	13	16	12	12	Bekaa	Shiite	Muslim

549	R1/-b1c	14	12	13	16	24	10	13	12	15	12	12	South	Shiite	Muslim
550	R1/-b1c	14	12	14	16	23	10	13	12	15	12	12	North	Shiite	Muslim
551	R1/-b1c	14	12	13	16	24	10	13	12	15	12	12	Beirut	Sunnite	Muslim
552	R1/-b1c	14	12	13	16	25	10	13	12	15	11	11	Mt Leb	Sunnite	Muslim
553	R1/-b1c	14	12	13	15	24	11	14	12	15	12	13	South	Sunnite	Muslim
554	R1/-b1c	14	12	13	18	24	10	12	12	15	12	13	South	Sunnite	Muslim
555	R1/-b1c	14	12	13	16	25	10	13	12	15	11	11	Mt Leb	Sunnite	Muslim
556	R1/-b1c	15	12	14	16	24	12	13	12	15	12	11	Beirut	Sunnite	Muslim
557	R1/-b1c	13	12	14	16	24	10	13	12	16	12	12	Beirut	Sunnite	Muslim
558	R1/-b1c	14	12	13	16	25	10	13	12	15	11	11	Mt Leb	Sunnite	Muslim
559	R1/-b1c	14	12	13	16	24	10	13	12	15	12	13	Bekaa	Sunnite	Muslim
560	R1/-b1c	14	12	13	16	25	10	13	12	15	11	11	Mt Leb	Sunnite	Muslim
561	R1a1	16	12	13	19	24	10	11	13	14	11	11	Mt Leb	Druze	Druze
562	R1a1	15	12	14	17	24	11	11	13	14	11	10	Mt Leb	Druze	Druze
563	R1a1	15	12	14	16	24	11	11	13	14	11	10	Mt Leb	Druze	Druze
564	R1a1	16	12	13	17	25	10	11	13	14	11	10	North	Maronite	Christian
565	R1a1	16	12	13	17	25	10	11	13	14	11	10	North	Maronite	Christian
566	R1a1	16	12	14	17	24	11	11	13	14	11	10	North	Maronite	Christian
567	R1a1	16	12	14	17	24	11	11	13	14	11	10	North	Orthodox	Christian
568	R1a1	15	13	14	18	25	10	11	13	14	11	11	Bekaa	Shiite	Muslim
569	R1a1	15	12	13	18	25	11	11	13	14	11	10	South	Shiite	Muslim
570	R1a1	16	12	12	17	25	11	11	13	14	11	13	South	Shiite	Muslim
571	R1a1	16	12	12	18	25	10	11	13	14	11	10	North	Sunnite	Muslim
572	R1a1	15	12	14	17	24	11	11	13	14	11	11	Beirut	Sunnite	Muslim
573	R1a1	14	12	14	18	24	11	10	13	14	11	10	Bekaa	Sunnite	Muslim
574	R1a1	15	12	12	16	24	10	11	13	14	11	11	North	Sunnite	Muslim
575	R1a1	17	12	13	19	25	10	11	13	15	11	10	North	Sunnite	Muslim
576	R2	14	12	14	15	23	11	10	14	16	11	11	Bekaa	Shiite	Muslim
577	R2	14	12	14	15	23	10	10	14	14	11	11	South	Sunnite	Muslim
578	–	14	12	13	16	25	10	13	12	15	11	11	Mt Leb	Druze	Druze
579	–	14	13	14	17	23	10	11	12	15	9	12	Mt Leb	Druze	Druze
580	–	14	12	14	17	23	10	14	13	14	11	12	North	Maronite	Christian
581	–	14	12	13	17	23	10	14	13	14	11	12	Mt Leb	Maronite	Christian
582	–	15	17	13	18	23	11	11	12	14	10	11	Bekaa	Shiite	Muslim
583	–	14	12	13	16	23	9	13	14	14	11	11	South	Shiite	Muslim
584	–	15	12	12	17	21	10	11	15	16	10	11	Mt Leb	Sunnite	Muslim
585	–	17	10	13	18	24	11	11	12	15	10	12	Bekaa	Sunnite	Muslim
586	–	15	11	13	17	21	10	11	12	16	10	12	North	Sunnite	Muslim
587	–	15	11	14	17	21	10	11	14	16	10	12	Beirut	Sunnite	Muslim
588	E*(xE1,E2,E3a,E3b)	.	.	.	.	.	.	.	.	.	.	.	Mt Leb	Druze	Druze
589	E*(xE1,E2,E3a,E3b)	.	.	.	.	.	.	.	.	.	.	.	Mt Leb	Maronite	Christian
590	E1	.	.	.	.	.	.	.	.	.	.	.	Beirut	Sunnite	Muslim
591	E3a	.	.	.	.	.	.	.	.	.	.	.	Beirut	Sunnite	Muslim
592	E3b1	.	.	.	.	.	.	.	.	.	.	.	North	Catholic	Christian
593	E3b1	.	.	.	.	.	.	.	.	.	.	.	Mt Leb	Druze	Druze
594	E3b1	.	.	.	.	.	.	.	.	.	.	.	Mt Leb	Druze	Druze
595	E3b1	.	.	.	.	.	.	.	.	.	.	.	Mt Leb	Druze	Druze
596	E3b1	.	.	.	.	.	.	.	.	.	.	.	Mt Leb	Druze	Druze
597	E3b1	.	.	.	.	.	.	.	.	.	.	.	Mt Leb	Druze	Druze
598	E3b1	.	.	.	.	.	.	.	.	.	.	.	Mt Leb	Druze	Druze
599	E3b1	.	.	.	.	.	.	.	.	.	.	.	Mt Leb	Druze	Druze
600	E3b1	.	.	.	.	.	.	.	.	.	.	.	Mt Leb	Druze	Druze
601	E3b1	.	.	.	.	.	.	.	.	.	.	.	Mt Leb	Druze	Druze

602	E3b1	.	.	.	.	.	.	.	.	.	.	.	Mt Leb	Druze	Druze
603	E3b1	.	.	.	.	.	.	.	.	.	.	.	Mt Leb	Druze	Druze
604	E3b1	.	.	.	.	.	.	.	.	.	.	.	Mt Leb	Druze	Druze
605	E3b1	.	.	.	.	.	.	.	.	.	.	.	Mt Leb	Druze	Druze
606	E3b1	.	.	.	.	.	.	.	.	.	.	.	Mt Leb	Druze	Druze
607	E3b1	.	.	.	.	.	.	.	.	.	.	.	North	Maronite	Christian
608	E3b1	.	.	.	.	.	.	.	.	.	.	.	North	Maronite	Christian
609	E3b1	.	.	.	.	.	.	.	.	.	.	.	South	Maronite	Christian
610	E3b1	.	.	.	.	.	.	.	.	.	.	.	Bekaa	Maronite	Christian
611	E3b1	.	.	.	.	.	.	.	.	.	.	.	South	Maronite	Christian
612	E3b1	.	.	.	.	.	.	.	.	.	.	.	North	Maronite	Christian
613	E3b1	.	.	.	.	.	.	.	.	.	.	.	South	Maronite	Christian
614	E3b1	.	.	.	.	.	.	.	.	.	.	.	North	Maronite	Christian
615	E3b1	.	.	.	.	.	.	.	.	.	.	.	North	Orthodox	Christian
616	E3b1	.	.	.	.	.	.	.	.	.	.	.	North	Orthodox	Christian
617	E3b1	.	.	.	.	.	.	.	.	.	.	.	Beirut	Orthodox	Christian
618	E3b1	.	.	.	.	.	.	.	.	.	.	.	North	Orthodox	Christian
619	E3b1	.	.	.	.	.	.	.	.	.	.	.	Mt Leb	Orthodox	Christian
620	E3b1	.	.	.	.	.	.	.	.	.	.	.	Mt Leb	Orthodox	Christian
621	E3b1	.	.	.	.	.	.	.	.	.	.	.	Lebanon	Shiite	Muslim
622	E3b1	.	.	.	.	.	.	.	.	.	.	.	South	Shiite	Muslim
623	E3b1	.	.	.	.	.	.	.	.	.	.	.	South	Shiite	Muslim
624	E3b1	.	.	.	.	.	.	.	.	.	.	.	South	Shiite	Muslim
625	E3b1	.	.	.	.	.	.	.	.	.	.	.	Bekaa	Shiite	Muslim
626	E3b1	.	.	.	.	.	.	.	.	.	.	.	North	Sunnite	Muslim
627	E3b1	.	.	.	.	.	.	.	.	.	.	.	North	Sunnite	Muslim
628	E3b1	.	.	.	.	.	.	.	.	.	.	.	North	Sunnite	Muslim
629	E3b1	.	.	.	.	.	.	.	.	.	.	.	Beirut	Sunnite	Muslim
630	E3b1	.	.	.	.	.	.	.	.	.	.	.	South	Sunnite	Muslim
631	E3b1	.	.	.	.	.	.	.	.	.	.	.	Beirut	Sunnite	Muslim
632	E3b1	.	.	.	.	.	.	.	.	.	.	.	Mt Leb	Sunnite	Muslim
633	E3b1	.	.	.	.	.	.	.	.	.	.	.	Beirut	Sunnite	Muslim
634	E3b1	.	.	.	.	.	.	.	.	.	.	.	North	Sunnite	Muslim
635	E3b2	.	.	.	.	.	.	.	.	.	.	.	Mt Leb	Druze	Druze
636	E3b2	.	.	.	.	.	.	.	.	.	.	.	Mt Leb	Druze	Druze
637	E3b2	.	.	.	.	.	.	.	.	.	.	.	Mt Leb	Druze	Druze
638	E3b2	.	.	.	.	.	.	.	.	.	.	.	North	Sunnite	Muslim
639	E3b3	.	.	.	.	.	.	.	.	.	.	.	Mt Leb	Druze	Druze
640	E3b3	.	.	.	.	.	.	.	.	.	.	.	Beirut	Greek Cath.	Christian
641	E3b3	.	.	.	.	.	.	.	.	.	.	.	Beirut	Maronite	Christian
642	E3b3	.	.	.	.	.	.	.	.	.	.	.	Mt Leb	Maronite	Christian
643	E3b3	.	.	.	.	.	.	.	.	.	.	.	Lebanon	Orthodox	Christian
644	E3b3	.	.	.	.	.	.	.	.	.	.	.	North	Orthodox	Christian
645	E3b3	.	.	.	.	.	.	.	.	.	.	.	Mt Leb	Shiite	Muslim
646	E3b3	.	.	.	.	.	.	.	.	.	.	.	South	Shiite	Muslim
647	E3b3	.	.	.	.	.	.	.	.	.	.	.	North	Sunnite	Muslim
648	E3b3	.	.	.	.	.	.	.	.	.	.	.	North	Sunnite	Muslim
649	E3b3	.	.	.	.	.	.	.	.	.	.	.	North	Sunnite	Muslim
650	E3b3	.	.	.	.	.	.	.	.	.	.	.	South	Sunnite	Muslim
651	E3b3	.	.	.	.	.	.	.	.	.	.	.	Beirut	Sunnite	Muslim
652	E3b3	.	.	.	.	.	.	.	.	.	.	.	South	Sunnite	Muslim
653	G	.	.	.	.	.	.	.	.	.	.	.	Beirut	Assyrian	Christian
654	G	.	.	.	.	.	.	.	.	.	.	.	Lebanon	Christian	Christian

655	G	.	.	.	.	.	.	.	.	.	.	.	Mt Leb	Druze	Druze
656	G	.	.	.	.	.	.	.	.	.	.	.	Bekaa	Maronite	Christian
657	G	.	.	.	.	.	.	.	.	.	.	.	Mt Leb	Maronite	Christian
658	G	.	.	.	.	.	.	.	.	.	.	.	Bekaa	Maronite	Christian
659	G	.	.	.	.	.	.	.	.	.	.	.	Mt Leb	Maronite	Christian
660	G	.	.	.	.	.	.	.	.	.	.	.	North	Maronite	Christian
661	G	.	.	.	.	.	.	.	.	.	.	.	South	Maronite	Christian
662	G	.	.	.	.	.	.	.	.	.	.	.	North	Maronite	Christian
663	G	.	.	.	.	.	.	.	.	.	.	.	Mt Leb	Orthodox	Christian
664	G	.	.	.	.	.	.	.	.	.	.	.	South	Orthodox	Christian
665	G	.	.	.	.	.	.	.	.	.	.	.	North	Orthodox	Christian
666	G	.	.	.	.	.	.	.	.	.	.	.	South	Shiite	Muslim
667	G	.	.	.	.	.	.	.	.	.	.	.	South	Shiite	Muslim
668	G	.	.	.	.	.	.	.	.	.	.	.	South	Shiite	Muslim
669	G	.	.	.	.	.	.	.	.	.	.	.	Bekaa	Shiite	Muslim
670	G	.	.	.	.	.	.	.	.	.	.	.	Bekaa	Shiite	Muslim
671	G	.	.	.	.	.	.	.	.	.	.	.	North	Sunnite	Muslim
672	G	.	.	.	.	.	.	.	.	.	.	.	Beirut	Sunnite	Muslim
673	G	.	.	.	.	.	.	.	.	.	.	.	Beirut	Sunnite	Muslim
674	G	.	.	.	.	.	.	.	.	.	.	.	Beirut	Sunnite	Muslim
675	I*(xI1a2,I1a3,I1b2,I1/-c)	.	.	.	.	.	.	.	.	.	.	.	South	Christian	Christian
676	I*(xI1a2,I1a3,I1b2,I1/-c)	.	.	.	.	.	.	.	.	.	.	.	Mt Leb	Druze	Druze
677	I*(xI1a2,I1a3,I1b2,I1/-c)	.	.	.	.	.	.	.	.	.	.	.	Mt Leb	Druze	Druze
678	I*(xI1a2,I1a3,I1b2,I1/-c)	.	.	.	.	.	.	.	.	.	.	.	Mt Leb	Druze	Druze
679	I*(xI1a2,I1a3,I1b2,I1/-c)	.	.	.	.	.	.	.	.	.	.	.	Mt Leb	Druze	Druze
680	I*(xI1a2,I1a3,I1b2,I1/-c)	.	.	.	.	.	.	.	.	.	.	.	Mt Leb	Druze	Druze
681	I*(xI1a2,I1a3,I1b2,I1/-c)	.	.	.	.	.	.	.	.	.	.	.	Mt Leb	Druze	Druze
682	I*(xI1a2,I1a3,I1b2,I1/-c)	.	.	.	.	.	.	.	.	.	.	.	Mt Leb	Druze	Druze
683	I*(xI1a2,I1a3,I1b2,I1/-c)	.	.	.	.	.	.	.	.	.	.	.	South	Maronite	Christian
684	I*(xI1a2,I1a3,I1b2,I1/-c)	.	.	.	.	.	.	.	.	.	.	.	Mt Leb	Maronite	Christian
685	I*(xI1a2,I1a3,I1b2,I1/-c)	.	.	.	.	.	.	.	.	.	.	.	Mt Leb	Orthodox	Christian
686	I*(xI1a2,I1a3,I1b2,I1/-c)	.	.	.	.	.	.	.	.	.	.	.	Mt Leb	Orthodox	Christian
687	I*(xI1a2,I1a3,I1b2,I1/-c)	.	.	.	.	.	.	.	.	.	.	.	North	Protestant	Christian
688	I*(xI1a2,I1a3,I1b2,I1/-c)	.	.	.	.	.	.	.	.	.	.	.	South	Sunnite	Muslim
689	I*(xI1a2,I1a3,I1b2,I1/-c)	.	.	.	.	.	.	.	.	.	.	.	Beirut	Sunnite	Muslim
690	I*(xI1a2,I1a3,I1b2,I1/-c)	.	.	.	.	.	.	.	.	.	.	.	North	Sunnite	Muslim
691	I*(xI1a2,I1a3,I1b2,I1/-c)	.	.	.	.	.	.	.	.	.	.	.	North	Sunnite	Muslim
692	I*(xI1a2,I1a3,I1b2,I1/-c)	.	.	.	.	.	.	.	.	.	.	.	North	Syriac Cath.	Christian
693	J* (xJ2)	.	.	.	.	.	.	.	.	.	.	.	Mt Leb	Druze	Druze
694	J* (xJ2)	.	.	.	.	.	.	.	.	.	.	.	Mt Leb	Druze	Druze
695	J* (xJ2)	.	.	.	.	.	.	.	.	.	.	.	South	Druze	Druze
696	J* (xJ2)	.	.	.	.	.	.	.	.	.	.	.	South	Druze	Druze
697	J* (xJ2)	.	.	.	.	.	.	.	.	.	.	.	Mt Leb	Druze	Druze
698	J* (xJ2)	.	.	.	.	.	.	.	.	.	.	.	Mt Leb	Druze	Druze
699	J* (xJ2)	.	.	.	.	.	.	.	.	.	.	.	Mt Leb	Druze	Druze
700	J* (xJ2)	.	.	.	.	.	.	.	.	.	.	.	Mt Leb	Druze	Druze
701	J* (xJ2)	.	.	.	.	.	.	.	.	.	.	.	Mt Leb	Druze	Druze
702	J* (xJ2)	.	.	.	.	.	.	.	.	.	.	.	Mt Leb	Druze	Druze
703	J* (xJ2)	.	.	.	.	.	.	.	.	.	.	.	Mt Leb	Druze	Druze
704	J* (xJ2)	.	.	.	.	.	.	.	.	.	.	.	Mt Leb	Druze	Druze
705	J* (xJ2)	.	.	.	.	.	.	.	.	.	.	.	Mt Leb	Druze	Druze
706	J* (xJ2)	.	.	.	.	.	.	.	.	.	.	.	Mt Leb	Druze	Druze
707	J* (xJ2)	.	.	.	.	.	.	.	.	.	.	.	Mt Leb	Maronite	Christian

708	J* (xJ2)	.	.	.	.	.	.	.	.	.	.	.	Beirut	Maronite	Christian
709	J* (xJ2)	.	.	.	.	.	.	.	.	.	.	.	North	Maronite	Christian
710	J* (xJ2)	.	.	.	.	.	.	.	.	.	.	.	Mt Leb	Maronite	Christian
711	J* (xJ2)	.	.	.	.	.	.	.	.	.	.	.	Mt Leb	Maronite	Christian
712	J* (xJ2)	.	.	.	.	.	.	.	.	.	.	.	Mt Leb	Maronite	Christian
713	J* (xJ2)	.	.	.	.	.	.	.	.	.	.	.	North	Maronite	Christian
714	J* (xJ2)	.	.	.	.	.	.	.	.	.	.	.	North	Maronite	Christian
715	J* (xJ2)	.	.	.	.	.	.	.	.	.	.	.	South	Maronite	Christian
716	J* (xJ2)	.	.	.	.	.	.	.	.	.	.	.	North	Maronite	Christian
717	J* (xJ2)	.	.	.	.	.	.	.	.	.	.	.	North	Maronite	Christian
718	J* (xJ2)	.	.	.	.	.	.	.	.	.	.	.	North	Maronite	Christian
719	J* (xJ2)	.	.	.	.	.	.	.	.	.	.	.	North	Maronite	Christian
720	J* (xJ2)	.	.	.	.	.	.	.	.	.	.	.	North	Maronite	Christian
721	J* (xJ2)	.	.	.	.	.	.	.	.	.	.	.	North	Maronite	Christian
722	J* (xJ2)	.	.	.	.	.	.	.	.	.	.	.	North	Maronite	Christian
723	J* (xJ2)	.	.	.	.	.	.	.	.	.	.	.	North	Maronite	Christian
724	J* (xJ2)	.	.	.	.	.	.	.	.	.	.	.	Mt Leb	Maronite	Christian
725	J* (xJ2)	.	.	.	.	.	.	.	.	.	.	.	North	Orthodox	Christian
726	J* (xJ2)	.	.	.	.	.	.	.	.	.	.	.	Mt Leb	Orthodox	Christian
727	J* (xJ2)	.	.	.	.	.	.	.	.	.	.	.	Bekaa	Orthodox	Christian
728	J* (xJ2)	.	.	.	.	.	.	.	.	.	.	.	South	Shiite	Muslim
729	J* (xJ2)	.	.	.	.	.	.	.	.	.	.	.	Bekaa	Shiite	Muslim
730	J* (xJ2)	.	.	.	.	.	.	.	.	.	.	.	North	Shiite	Muslim
731	J* (xJ2)	.	.	.	.	.	.	.	.	.	.	.	Bekaa	Shiite	Muslim
732	J* (xJ2)	.	.	.	.	.	.	.	.	.	.	.	South	Shiite	Muslim
733	J* (xJ2)	.	.	.	.	.	.	.	.	.	.	.	South	Shiite	Muslim
734	J* (xJ2)	.	.	.	.	.	.	.	.	.	.	.	South	Shiite	Muslim
735	J* (xJ2)	.	.	.	.	.	.	.	.	.	.	.	South	Shiite	Muslim
736	J* (xJ2)	.	.	.	.	.	.	.	.	.	.	.	South	Shiite	Muslim
737	J* (xJ2)	.	.	.	.	.	.	.	.	.	.	.	South	Shiite	Muslim
738	J* (xJ2)	.	.	.	.	.	.	.	.	.	.	.	Bekaa	Shiite	Muslim
739	J* (xJ2)	.	.	.	.	.	.	.	.	.	.	.	South	Shiite	Muslim
740	J* (xJ2)	.	.	.	.	.	.	.	.	.	.	.	Bekaa	Shiite	Muslim
741	J* (xJ2)	.	.	.	.	.	.	.	.	.	.	.	South	Shiite	Muslim
742	J* (xJ2)	.	.	.	.	.	.	.	.	.	.	.	North	Sunnite	Muslim
743	J* (xJ2)	.	.	.	.	.	.	.	.	.	.	.	Bekaa	Sunnite	Muslim
744	J* (xJ2)	.	.	.	.	.	.	.	.	.	.	.	North	Sunnite	Muslim
745	J* (xJ2)	.	.	.	.	.	.	.	.	.	.	.	Beirut	Sunnite	Muslim
746	J* (xJ2)	.	.	.	.	.	.	.	.	.	.	.	Mt Leb	Sunnite	Muslim
747	J* (xJ2)	.	.	.	.	.	.	.	.	.	.	.	North	Sunnite	Muslim
748	J* (xJ2)	.	.	.	.	.	.	.	.	.	.	.	North	Sunnite	Muslim
749	J* (xJ2)	.	.	.	.	.	.	.	.	.	.	.	North	Sunnite	Muslim
750	J* (xJ2)	.	.	.	.	.	.	.	.	.	.	.	North	Sunnite	Muslim
751	J* (xJ2)	.	.	.	.	.	.	.	.	.	.	.	North	Sunnite	Muslim
752	J* (xJ2)	.	.	.	.	.	.	.	.	.	.	.	South	Sunnite	Muslim
753	J* (xJ2)	.	.	.	.	.	.	.	.	.	.	.	Beirut	Sunnite	Muslim
754	J* (xJ2)	.	.	.	.	.	.	.	.	.	.	.	Beirut	Sunnite	Muslim
755	J* (xJ2)	.	.	.	.	.	.	.	.	.	.	.	North	Sunnite	Muslim
756	J* (xJ2)	.	.	.	.	.	.	.	.	.	.	.	North	Sunnite	Muslim
757	J2/-f1*(xJ2f1a)	.	.	.	.	.	.	.	.	.	.	.	Mt Leb	Maronite	Christian
758	J2/-f1*(xJ2f1a)	.	.	.	.	.	.	.	.	.	.	.	Mt Leb	Maronite	Christian
759	J2/-f1*(xJ2f1a)	.	.	.	.	.	.	.	.	.	.	.	North	Maronite	Christian
760	J2/-f1*(xJ2f1a)	.	.	.	.	.	.	.	.	.	.	.	Mt Leb	Maronite	Christian

761	J2/-f1*(xJ2f1a)	.	.	.	.	.	.	.	.	.	.	.	North	Maronite	Christian
762	J2/-f1*(xJ2f1a)	.	.	.	.	.	.	.	.	.	.	.	North	Maronite	Christian
763	J2/-f1*(xJ2f1a)	.	.	.	.	.	.	.	.	.	.	.	North	Maronite	Christian
764	J2/-f1*(xJ2f1a)	.	.	.	.	.	.	.	.	.	.	.	North	Maronite	Christian
765	J2/-f1*(xJ2f1a)	.	.	.	.	.	.	.	.	.	.	.	Mt Leb	Maronite	Christian
766	J2/-f1*(xJ2f1a)	.	.	.	.	.	.	.	.	.	.	.	North	Maronite	Christian
767	J2/-f1*(xJ2f1a)	.	.	.	.	.	.	.	.	.	.	.	North	Maronite	Christian
768	J2/-f1*(xJ2f1a)	.	.	.	.	.	.	.	.	.	.	.	North	Maronite	Christian
769	J2/-f1*(xJ2f1a)	.	.	.	.	.	.	.	.	.	.	.	North	Maronite	Christian
770	J2/-f1*(xJ2f1a)	.	.	.	.	.	.	.	.	.	.	.	Mt Leb	Maronite	Christian
771	J2/-f1*(xJ2f1a)	.	.	.	.	.	.	.	.	.	.	.	North	Maronite	Christian
772	J2/-f1*(xJ2f1a)	.	.	.	.	.	.	.	.	.	.	.	North	Maronite	Christian
773	J2/-f1*(xJ2f1a)	.	.	.	.	.	.	.	.	.	.	.	Mt Leb	Maronite	Christian
774	J2/-f1*(xJ2f1a)	.	.	.	.	.	.	.	.	.	.	.	Mt Leb	Maronite	Christian
775	J2/-f1*(xJ2f1a)	.	.	.	.	.	.	.	.	.	.	.	North	Orthodox	Christian
776	J2/-f1*(xJ2f1a)	.	.	.	.	.	.	.	.	.	.	.	South	Shiite	Muslim
777	J2/-f1*(xJ2f1a)	.	.	.	.	.	.	.	.	.	.	.	South	Shiite	Muslim
778	J2/-f1*(xJ2f1a)	.	.	.	.	.	.	.	.	.	.	.	South	Shiite	Muslim
779	J2/-f1*(xJ2f1a)	.	.	.	.	.	.	.	.	.	.	.	North	Sunnite	Muslim
780	J2/-f1*(xJ2f1a)	.	.	.	.	.	.	.	.	.	.	.	South	Sunnite	Muslim
781	J2/-f1*(xJ2f1a)	.	.	.	.	.	.	.	.	.	.	.	Beirut	Sunnite	Muslim
782	J2/-f1*(xJ2f1a)	.	.	.	.	.	.	.	.	.	.	.	North	Sunnite	Muslim
783	J2a	.	.	.	.	.	.	.	.	.	.	.	North	Catholic	Christian
784	J2a	.	.	.	.	.	.	.	.	.	.	.	Beirut	Catholic	Christian
785	J2a	.	.	.	.	.	.	.	.	.	.	.	Bekaa	Catholic	Christian
786	J2a	.	.	.	.	.	.	.	.	.	.	.	Bekaa	Catholic	Christian
787	J2a	.	.	.	.	.	.	.	.	.	.	.	Mt Leb	Catholic	Christian
788	J2a	.	.	.	.	.	.	.	.	.	.	.	Mt Leb	Druze	Druze
789	J2a	.	.	.	.	.	.	.	.	.	.	.	Mt Leb	Druze	Druze
790	J2a	.	.	.	.	.	.	.	.	.	.	.	Mt Leb	Druze	Druze
791	J2a	.	.	.	.	.	.	.	.	.	.	.	South	Druze	Muslim
792	J2a	.	.	.	.	.	.	.	.	.	.	.	Mt Leb	Druze	Druze
793	J2a	.	.	.	.	.	.	.	.	.	.	.	Mt Leb	Druze	Druze
794	J2a	.	.	.	.	.	.	.	.	.	.	.	Mt Leb	Druze	Druze
795	J2a	.	.	.	.	.	.	.	.	.	.	.	Mt Leb	Druze	Druze
796	J2a	.	.	.	.	.	.	.	.	.	.	.	Mt Leb	Druze	Druze
797	J2a	.	.	.	.	.	.	.	.	.	.	.	Mt Leb	Druze	Druze
798	J2a	.	.	.	.	.	.	.	.	.	.	.	Mt Leb	Druze	Druze
799	J2a	.	.	.	.	.	.	.	.	.	.	.	Mt Leb	Druze	Druze
800	J2a	.	.	.	.	.	.	.	.	.	.	.	Mt Leb	Druze	Druze
801	J2a	.	.	.	.	.	.	.	.	.	.	.	Mt Leb	Druze	Druze
802	J2a	.	.	.	.	.	.	.	.	.	.	.	North	Maronite	Christian
803	J2a	.	.	.	.	.	.	.	.	.	.	.	North	Maronite	Christian
804	J2a	.	.	.	.	.	.	.	.	.	.	.	Mt Leb	Maronite	Christian
805	J2a	.	.	.	.	.	.	.	.	.	.	.	South	Maronite	Christian
806	J2a	.	.	.	.	.	.	.	.	.	.	.	Mt Leb	Maronite	Christian
807	J2a	.	.	.	.	.	.	.	.	.	.	.	Bekaa	Maronite	Christian
808	J2a	.	.	.	.	.	.	.	.	.	.	.	Beirut	Maronite	Christian
809	J2a	.	.	.	.	.	.	.	.	.	.	.	North	Maronite	Christian
810	J2a	.	.	.	.	.	.	.	.	.	.	.	Mt Leb	Maronite	Christian
811	J2a	.	.	.	.	.	.	.	.	.	.	.	North	Maronite	Christian
812	J2a	.	.	.	.	.	.	.	.	.	.	.	Mt Leb	Maronite	Christian
813	J2a	.	.	.	.	.	.	.	.	.	.	.	North	Maronite	Christian

814	J2a	.	.	.	.	.	.	.	.	.	.	.	South	Muslim	Muslim
815	J2a	.	.	.	.	.	.	.	.	.	.	.	North	Orthodox	Christian
816	J2a	.	.	.	.	.	.	.	.	.	.	.	Mt Leb	Orthodox	Christian
817	J2a	.	.	.	.	.	.	.	.	.	.	.	Bekaa	Orthodox	Christian
818	J2a	.	.	.	.	.	.	.	.	.	.	.	North	Orthodox	Christian
819	J2a	.	.	.	.	.	.	.	.	.	.	.	South	Shiite	Muslim
820	J2a	.	.	.	.	.	.	.	.	.	.	.	Lebanon	Shiite	Muslim
821	J2a	.	.	.	.	.	.	.	.	.	.	.	Mt Leb	Shiite	Muslim
822	J2a	.	.	.	.	.	.	.	.	.	.	.	South	Shiite	Muslim
823	J2a	.	.	.	.	.	.	.	.	.	.	.	South	Shiite	Muslim
824	J2a	.	.	.	.	.	.	.	.	.	.	.	South	Shiite	Muslim
825	J2a	.	.	.	.	.	.	.	.	.	.	.	North	Sunnite	Muslim
826	J2a	.	.	.	.	.	.	.	.	.	.	.	North	Sunnite	Muslim
827	J2a	.	.	.	.	.	.	.	.	.	.	.	North	Sunnite	Muslim
828	J2a	.	.	.	.	.	.	.	.	.	.	.	Beirut	Sunnite	Muslim
829	J2a	.	.	.	.	.	.	.	.	.	.	.	North	Sunnite	Muslim
830	J2a	.	.	.	.	.	.	.	.	.	.	.	North	Sunnite	Muslim
831	J2a	.	.	.	.	.	.	.	.	.	.	.	South	Sunnite	Muslim
832	J2a	.	.	.	.	.	.	.	.	.	.	.	South	Sunnite	Muslim
833	J2a	.	.	.	.	.	.	.	.	.	.	.	South	Sunnite	Muslim
834	J2a	.	.	.	.	.	.	.	.	.	.	.	North	Sunnite	Muslim
835	J2a	.	.	.	.	.	.	.	.	.	.	.	South	Sunnite	Muslim
836	J2a	.	.	.	.	.	.	.	.	.	.	.	Beirut	Syriac Cath.	Christian
837	J2e*(xJ2e1)	.	.	.	.	.	.	.	.	.	.	.	Mt Leb	Maronite	Christian
838	J2e*(xJ2e1)	.	.	.	.	.	.	.	.	.	.	.	North	Maronite	Christian
839	J2e*(xJ2e1)	.	.	.	.	.	.	.	.	.	.	.	Mt Leb	Maronite	Christian
840	J2e*(xJ2e1)	.	.	.	.	.	.	.	.	.	.	.	North	Maronite	Christian
841	J2e*(xJ2e1)	.	.	.	.	.	.	.	.	.	.	.	North	Orthodox	Christian
842	J2e*(xJ2e1)	.	.	.	.	.	.	.	.	.	.	.	Mt Leb	Orthodox	Christian
843	J2e*(xJ2e1)	.	.	.	.	.	.	.	.	.	.	.	North	Orthodox	Christian
844	J2e*(xJ2e1)	.	.	.	.	.	.	.	.	.	.	.	North	Sunnite	Muslim
845	K*(xK2,K3,L,M,NO,P)	.	.	.	.	.	.	.	.	.	.	.	North	Maronite	Christian
846	K*(xK2,K3,L,M,NO,P)	.	.	.	.	.	.	.	.	.	.	.	Mt Leb	Maronite	Christian
847	K*(xK2,K3,L,M,NO,P)	.	.	.	.	.	.	.	.	.	.	.	North	Maronite	Christian
848	K*(xK2,K3,L,M,NO,P)	.	.	.	.	.	.	.	.	.	.	.	North	Sunnite	Muslim
849	K2	.	.	.	.	.	.	.	.	.	.	.	Bekaa	Catholic	Christian
850	K2	.	.	.	.	.	.	.	.	.	.	.	Mt Leb	Druze	Druze
851	K2	.	.	.	.	.	.	.	.	.	.	.	Mt Leb	Druze	Druze
852	K2	.	.	.	.	.	.	.	.	.	.	.	Mt Leb	Druze	Druze
853	K2	.	.	.	.	.	.	.	.	.	.	.	Mt Leb	Druze	Druze
854	K2	.	.	.	.	.	.	.	.	.	.	.	Mt Leb	Druze	Druze
855	K2	.	.	.	.	.	.	.	.	.	.	.	Mt Leb	Druze	Druze
856	K2	.	.	.	.	.	.	.	.	.	.	.	Mt Leb	Druze	Druze
857	K2	.	.	.	.	.	.	.	.	.	.	.	Mt Leb	Druze	Druze
858	K2	.	.	.	.	.	.	.	.	.	.	.	North	Maronite	Christian
859	K2	.	.	.	.	.	.	.	.	.	.	.	North	Maronite	Christian
860	K2	.	.	.	.	.	.	.	.	.	.	.	Mt Leb	Maronite	Christian
861	K2	.	.	.	.	.	.	.	.	.	.	.	North	Maronite	Christian
862	K2	.	.	.	.	.	.	.	.	.	.	.	Beirut	Orthodox	Christian
863	K2	.	.	.	.	.	.	.	.	.	.	.	South	Shiite	Muslim
864	K2	.	.	.	.	.	.	.	.	.	.	.	South	Shiite	Muslim
865	K2	.	.	.	.	.	.	.	.	.	.	.	Beirut	Sunnite	Muslim
866	K2	.	.	.	.	.	.	.	.	.	.	.	Beirut	Sunnite	Muslim

867	K2	.	.	.	.	.	.	.	.	.	.	.	North	Sunnite	Muslim
868	K2	.	.	.	.	.	.	.	.	.	.	.	South	Sunnite	Muslim
869	L*	.	.	.	.	.	.	.	.	.	.	.	Mt Leb	Druze	Druze
870	L*	.	.	.	.	.	.	.	.	.	.	.	Mt Leb	Druze	Druze
871	L*	.	.	.	.	.	.	.	.	.	.	.	Mt Leb	Druze	Druze
872	L*	.	.	.	.	.	.	.	.	.	.	.	Mt Leb	Druze	Druze
873	L*	.	.	.	.	.	.	.	.	.	.	.	Mt Leb	Druze	Druze
874	L*	.	.	.	.	.	.	.	.	.	.	.	Mt Leb	Druze	Druze
875	L*	.	.	.	.	.	.	.	.	.	.	.	Mt Leb	Druze	Druze
876	L*	.	.	.	.	.	.	.	.	.	.	.	North	Maronite	Christian
877	L*	.	.	.	.	.	.	.	.	.	.	.	North	Maronite	Christian
878	L*	.	.	.	.	.	.	.	.	.	.	.	South	Maronite	Christian
879	L*	.	.	.	.	.	.	.	.	.	.	.	Mt Leb	Maronite	Christian
880	L*	.	.	.	.	.	.	.	.	.	.	.	North	Maronite	Christian
881	L*	.	.	.	.	.	.	.	.	.	.	.	South	Maronite	Christian
882	L*	.	.	.	.	.	.	.	.	.	.	.	Mt Leb	Orthodox	Christian
883	L*	.	.	.	.	.	.	.	.	.	.	.	South	Shiite	Muslim
884	Q*	.	.	.	.	.	.	.	.	.	.	.	Beirut	Arm. Orth.	Christian
885	Q*	.	.	.	.	.	.	.	.	.	.	.	South	Druze	Druze
886	Q*	.	.	.	.	.	.	.	.	.	.	.	Mt Leb	Evangelical	Christian
887	Q*	.	.	.	.	.	.	.	.	.	.	.	Bekaa	Orthodox	Christian
888	Q*	.	.	.	.	.	.	.	.	.	.	.	North	Orthodox	Christian
889	Q2	.	.	.	.	.	.	.	.	.	.	.	South	Shiite	Muslim
890	Q2	.	.	.	.	.	.	.	.	.	.	.	North	Sunnite	Muslim
891	R*	.	.	.	.	.	.	.	.	.	.	.	South	Druze	Druze
892	R*	.	.	.	.	.	.	.	.	.	.	.	North	Maronite	Christian
893	R1/-b*	.	.	.	.	.	.	.	.	.	.	.	South	Shiite	Muslim
894	R1/-b1a	.	.	.	.	.	.	.	.	.	.	.	Mt Leb	Druze	Druze
895	R1/-b1a	.	.	.	.	.	.	.	.	.	.	.	South	Sunnite	Muslim
896	R1/-b1c	.	.	.	.	.	.	.	.	.	.	.	Bekaa	Catholic	Christian
897	R1/-b1c	.	.	.	.	.	.	.	.	.	.	.	South	Catholic	Christian
898	R1/-b1c	.	.	.	.	.	.	.	.	.	.	.	Mt Leb	Druze	Druze
899	R1/-b1c	.	.	.	.	.	.	.	.	.	.	.	Mt Leb	Druze	Druze
900	R1/-b1c	.	.	.	.	.	.	.	.	.	.	.	Mt Leb	Druze	Druze
901	R1/-b1c	.	.	.	.	.	.	.	.	.	.	.	Mt Leb	Druze	Druze
902	R1/-b1c	.	.	.	.	.	.	.	.	.	.	.	Mt Leb	Druze	Druze
903	R1/-b1c	.	.	.	.	.	.	.	.	.	.	.	Mt Leb	Druze	Druze
904	R1/-b1c	.	.	.	.	.	.	.	.	.	.	.	Mt Leb	Druze	Druze
905	R1/-b1c	.	.	.	.	.	.	.	.	.	.	.	Bekaa	Druze	Druze
906	R1/-b1c	.	.	.	.	.	.	.	.	.	.	.	Mt Leb	Druze	Druze
907	R1/-b1c	.	.	.	.	.	.	.	.	.	.	.	Mt Leb	Maronite	Christian
908	R1/-b1c	.	.	.	.	.	.	.	.	.	.	.	South	Maronite	Christian
909	R1/-b1c	.	.	.	.	.	.	.	.	.	.	.	North	Maronite	Christian
910	R1/-b1c	.	.	.	.	.	.	.	.	.	.	.	North	Maronite	Christian
911	R1/-b1c	.	.	.	.	.	.	.	.	.	.	.	North	Maronite	Christian
912	R1/-b1c	.	.	.	.	.	.	.	.	.	.	.	North	Maronite	Christian
913	R1/-b1c	.	.	.	.	.	.	.	.	.	.	.	South	Maronite	Christian
914	R1/-b1c	.	.	.	.	.	.	.	.	.	.	.	Mt Leb	Maronite	Christian
915	R1/-b1c	.	.	.	.	.	.	.	.	.	.	.	Mt Leb	Maronite	Christian
916	R1/-b1c	.	.	.	.	.	.	.	.	.	.	.	North	Orthodox	Christian
917	R1/-b1c	.	.	.	.	.	.	.	.	.	.	.	Lebanon	Shiite	Muslim
918	R1/-b1c	.	.	.	.	.	.	.	.	.	.	.	Bekaa	Sunnite	Muslim
919	R1a1	.	.	.	.	.	.	.	.	.	.	.	North	Maronite	Christian

920	R1a1	.	.	.	.	.	.	.	.	.	.	.	South	Orthodox	Christian
921	R1a1	.	.	.	.	.	.	.	.	.	.	.	North	Orthodox	Christian
922	R1a1	.	.	.	.	.	.	.	.	.	.	.	Mt Leb	Shiite	Muslim
923	R1a1	.	.	.	.	.	.	.	.	.	.	.	South	Shiite	Muslim
924	R1a1	.	.	.	.	.	.	.	.	.	.	.	North	Sunnite	Muslim
925	R1a1	.	.	.	.	.	.	.	.	.	.	.	South	Sunnite	Muslim
926	R1a1	.	.	.	.	.	.	.	.	.	.	.	Beirut	Sunnite	Muslim
927	–	.	.	.	.	.	.	.	.	.	.	.	Mt Leb	Druze	Druze
928	–	.	.	.	.	.	.	.	.	.	.	.	Mt Leb	Maronite	Christian
929	–	.	.	.	.	.	.	.	.	.	.	.	Lebanon	Shiite	Muslim
930	–	.	.	.	.	.	.	.	.	.	.	.	South	Shiite	Muslim
931	–	.	.	.	.	.	.	.	.	.	.	.	Bekaa	Sunnite	Muslim
932	–	.	.	.	.	.	.	.	.	.	.	.	South	Sunnite	Muslim
933	–	.	.	.	.	.	.	.	.	.	.	.	Lebanon	Sunnite	Muslim
934	–	.	.	.	.	.	.	.	.	.	.	.	Beirut	Sunnite	Muslim
935	–	.	.	.	.	.	.	.	.	.	.	.	North	Sunnite	Muslim

Table S2. Genographic Public Participation data used in this work.

**Y Chromosome Haplogroup/SNP Marker**	**Father's Place of Birth**	**STR Marker**										
		19	385 loci	388	389I	389b	390	391	392	393	426	439
Haplogroup E3b/M35	Oman	13	16-17	12	13	17	25	10	11	13	11	11
Haplogroup K/M9	Oman	14	14-16	13	13	16	23	10	13	13	11	11
Haplogroup J*(xJ2)/12f2.1	Qatar	14	13-19	17	13	17	23	10	11	12	11	11
Haplogroup R1a/10831.2	Qatar	16	11-14	12	13	16	26	11	11	13	12	10
Haplogroup E3b/M35	Saudi Arabia	14	17-18	12	12	17	23	10	11	13	11	10
Haplogroup J*(xJ2)/12f2.1	Saudi Arabia	14	13-19	17	13	18	23	11	11	12	11	11
Haplogroup J1/M267	Saudi Arabia	14	13-19	16	13	17	23	10	11	12	11	13
Haplogroup Q/M242	Saudi Arabia	14	15-16	12	13	16	22	10	15	13	12	12
Haplogroup R1a1/M17	Saudi Arabia	14	11-14	12	14	17	25	10	11	13	12	10
Haplogroup J*(xJ2)/12f2.1	United Arab Emirates	14	13-19	17	14	18	23	11	11	12	11	11
Haplogroup J2/M172	United Arab Emirates	14	14-18	15	13	16	22	10	11	13	11	11
Haplogroup E3b/M35	Yemen	14	16-17	12	13	17	23	10	11	13	11	12
Haplogroup J1/M267	Yemen	14	13-15	16	13	17	23	11	11	12	11	13
Haplogroup J2/M172	Yemen	13	14-17	15	12	17	24	10	11	12	11	12

Y Chromosome Haplogroup/SNP Marker	Father's Ethnicity	Father's Place of Birth	STR Marker										
			19	385 loci	388	389I	389b	390	391	392	393	426	439
Haplogroup I/M170	Breton	France	16	15-16	15	13	16	23	11	12	14	11	11
Haplogroup I/M170	Breton/Normand	France	16	12-18	13	13	17	26	11	11	13	11	11
Haplogroup I/M170	french	France	15	14-15	13	12	17	23	10	11	14	11	10
Haplogroup I/M170	french (Burgundy)	France	16	14-15	13	13	17	24	10	11	13	11	12
Haplogroup I/M170	French	France	16	14-14	13	12	17	23	10	12	15	11	12
Haplogroup I/M170	French	France	15	16-18	13	13	18	22	10	12	14	11	12
Haplogroup I/M170	French	France	15	15-17	13	14	17	22	10	12	14	11	11
Haplogroup I/M170	French	France	14	13-14	14	12	16	23	10	11	13	11	11
Haplogroup I/M170	French (Breton)	France	16	15-16	13	13	16	23	10	12	14	11	11
Haplogroup I/M170	French (east of France)	France	14	12-14	13	13	16	24	10	11	14	12	10
Haplogroup I/M170	French catholic	France	14	13-13	14	12	16	22	10	11	13	11	11
Haplogroup I/M170	FRENCH	France	17	12-12	13	13	15	23	10	11	13	11	13
Haplogroup I/M170	FRENCH	France	15	13-14	14	12	16	23	10	11	13	11	11
Haplogroup I/M170	Little Brittany	France	16	14-15	13	13	16	23	11	12	14	11	11
Haplogroup I/M170	FRENCH	France	15	15-15	13	14	18	23	10	12	14	11	11
Haplogroup I/M170	East Prussian German	Germany	14	14-15	14	12	16	22	10	11	13	11	11
Haplogroup I/M170	german	Germany	15	14-15	13	14	18	23	10	12	14	11	11
Haplogroup I/M170	german	Germany	17	13-15	14	14	18	23	10	12	16	11	12
Haplogroup I/M170	german	Germany	14	15-16	13	14	17	23	10	12	15	11	12
Haplogroup I/M170	german	Germany	14	13-13	16	12	16	22	10	11	14	11	13
Haplogroup I/M170	german	Germany	14	13-15	14	12	16	22	10	11	13	11	11
Haplogroup I/M170	german	Germany	16	15-15	13	13	18	25	11	11	13	11	13
Haplogroup I/M170	german	Germany	14	13-14	14	12	16	23	10	11	15	11	12
Haplogroup I/M170	german	Germany	15	14-15	13	14	18	23	10	12	14	11	11
Haplogroup I/M170	german catholic	Germany	17	14-15	13	13	17	24	11	11	13	11	13
Haplogroup I/M170	german lutheran	Germany	14	14-14	14	12	16	22	11	11	13	11	11
Haplogroup I/M170	german protestant	Germany	16	15-16	13	14	17	22	10	12	15	11	11
Haplogroup I/M170	German	Germany	14	13-14	13	12	17	22	10	11	13	11	11
Haplogroup I/M170	German	Germany	15	15-15	13	13	18	20	10	12	14	11	12
Haplogroup I/M170	German	Germany	17	16-16	14	12	16	25	11	11	13	11	11
Haplogroup I/M170	German	Germany	15	13-14	15	12	16	22	10	11	14	11	12
Haplogroup I/M170	German	Germany	14	14-14	15	13	16	22	10	11	13	11	11
Haplogroup I/M170	German	Germany	15	15-15	13	14	17	23	10	12	15	11	11

Haplogroup I/M170	German	Germany	14	13-14	14	12	17	22	10	11	13	11	11
Haplogroup I/M170	German	Germany	14	14-14	14	12	16	22	10	11	14	11	13
Haplogroup I/M170	German	Germany	14	13-14	14	13	16	22	10	11	13	11	12
Haplogroup I/M170	German	Germany	14	13-13	14	12	16	22	10	11	13	11	11
Haplogroup I/M170	German	Germany	15	15-16	13	13	19	23	10	12	14	11	11
Haplogroup I/M170	German	Germany	14	13-14	14	12	16	23	10	11	13	11	11
Haplogroup I/M170	German	Germany	15	14-15	10	12	16	22	10	11	13	11	12
Haplogroup I/M170	German	Germany	14	13-14	14	12	17	22	10	11	13	11	12
Haplogroup I/M170	German	Germany	14	14-14	14	12	16	22	10	11	12	11	11
Haplogroup I/M170	German	Germany	14	13-14	14	12	16	22	11	11	13	11	12
Haplogroup I/M170	German	Germany	14	13-14	14	12	16	22	10	11	12	11	12
Haplogroup I/M170	German	Germany	14	13-14	14	12	18	21	10	11	14	11	11
Haplogroup I/M170	German	Germany	14	13-14	14	12	16	22	10	11	14	11	12
Haplogroup I/M170	German	Germany	16	15-16	13	13	16	23	11	12	15	11	11
Haplogroup I/M170	German	Germany	14	13-14	14	12	16	22	10	11	13	11	12
Haplogroup I/M170	German	Germany	16	13-16	13	13	17	25	11	11	13	11	11
Haplogroup I/M170	German	Germany	15	15-15	13	14	19	23	10	12	14	11	11
Haplogroup I/M170	German	Germany	14	13-14	14	12	16	22	10	11	14	11	11
Haplogroup I/M170	German	Germany	14	14-14	14	12	16	24	10	11	13	11	12
Haplogroup I/M170	German	Germany	14	13-14	12	13	16	22	10	11	13	11	12
Haplogroup I/M170	German	Germany	14	13-14	14	12	16	22	10	11	13	11	11
Haplogroup I/M170	German	Germany	14	13-14	14	12	16	22	11	11	13	11	11
Haplogroup I/M170	German	Germany	15	13-14	14	12	16	22	10	11	13	11	11
Haplogroup I/M170	German	Germany	15	10-15	13	14	18	23	10	12	15	11	11
Haplogroup I/M170	German	Germany	17	12-13	13	13	18	24	10	11	14	11	11
Haplogroup I/M170	German	Germany	14	14-14	14	12	16	23	10	11	13	11	11
Haplogroup I/M170	German	Germany	14	13-14	14	12	16	22	10	11	13	11	11
Haplogroup I/M170	German	Germany	16	17-17	13	13	16	23	10	12	15	11	11
Haplogroup I/M170	German	Germany	15	14-15	13	14	19	23	10	12	13	11	11
Haplogroup I/M170	German	Germany	14	13-14	14	12	17	22	10	11	13	10	11
Haplogroup I/M170	German	Germany	14	15-17	13	13	17	24	10	12	15	11	13
Haplogroup I/M170	German	Germany	15	15-17	13	14	18	23	11	12	14	11	11
Haplogroup I/M170	German	Germany	15	14-15	13	14	19	23	10	12	13	11	11
Haplogroup I/M170	German Christians	Germany	14	13-15	14	12	16	23	10	11	13	11	11
Haplogroup I/M170	German Lutheran	Germany	14	13-14	14	12	16	22	10	11	13	11	11
Haplogroup I/M170	German protestant	Germany	15	15-15	13	14	17	23	10	12	15	11	11
Haplogroup I/M170	Germany	Germany	16	16-16	13	14	16	24	10	12	15	11	11
Haplogroup I/M170	Northern Garmany Lutheran	Germany	16	15-16	12	14	17	23	10	12	15	11	11

Haplogroup I/M170	Protestant German	Germany	15	13-14	14	12	16	22	11	11	13	11	12
Haplogroup I/M170	german	Germany	13	13-14	14	12	17	22	10	11	13	11	11
Haplogroup I/M170	German	Germany	14	13-14	15	12	16	23	9	11	13	11	11
Haplogroup I/M170	German	Germany	15	13-14	14	12	16	22	10	11	12	11	11
Haplogroup I/M170	German	Germany	14	13-14	14	12	16	23	10	11	13	11	11
Haplogroup I/M170	German	Germany	16	15-15	13	14	17	22	10	12	15	11	11
Haplogroup I/M170	German	Germany	14	13-13	14	12	16	22	10	11	13	11	11
Haplogroup I/M170	German	Germany	14	13-14	14	12	16	23	10	11	13	11	11
Haplogroup I/M170	German	Germany	14	12-13	14	12	17	22	10	11	13	11	10
Haplogroup I/M170	German	Germany	15	13-14	14	12	16	22	10	11	13	11	11
Haplogroup I/M170	German	Germany	15	15-15	13	14	17	22	10	13	15	11	11
Haplogroup I/M170	italian	Italy	16	12-14	13	12	17	22	10	11	12	11	11
Haplogroup I/M170	italian	Italy	16	12-14	13	14	16	24	10	11	12	11	11
Haplogroup I/M170	italian	Italy	14	13-14	14	12	16	23	10	11	13	11	11
Haplogroup I/M170	italian	Italy	15	14-15	13	13	18	24	11	11	13	11	12
Haplogroup I/M170	Italian	Italy	15	13-18	13	14	17	23	10	12	14	11	11
Haplogroup I/M170	Italian	Italy	15	13-16	13	13	18	23	10	12	12	11	11
Haplogroup I/M170	Italian	Italy	15	14-14	14	12	16	23	10	11	13	11	11
Haplogroup I/M170	Italian	Italy	15	13-17	13	12	18	25	11	11	13	11	11
Haplogroup I/M170	Italian	Italy	16	12-15	13	12	17	22	9	11	12	11	11
Haplogroup I/M170	Italian	Italy	16	15-15	13	13	16	23	11	12	13	11	13
Haplogroup I/M170	Italian	Italy	16	13-16	13	12	16	25	10	11	13	11	11
Haplogroup I/M170	Italian	Italy	16	14-17	13	14	18	23	10	12	14	11	12
Haplogroup I/M170	Italian	Italy	14	13-14	14	12	16	23	10	11	13	11	11
Haplogroup I/M170	Italian	Italy	17	12-12	13	14	15	23	10	11	13	11	13
Haplogroup I/M170	Italian	Italy	15	15-15	13	13	18	24	10	12	14	11	12
Haplogroup I/M170	Italian	Italy	16	14-15	13	13	17	24	11	11	13	11	12
Haplogroup I/M170	Italian Catholic	Italy	15	15-16	13	14	16	23	10	12	15	11	11
Haplogroup I/M170	Italy	Italy	14	13-14	14	12	16	23	11	11	13	11	10
Haplogroup I/M170	ITALIAN	Italy	15	15-15	13	13	17	23	10	12	14	11	11
Haplogroup I/M170	ITALIAN	Italy	14	12-13	15	12	16	22	10	11	13	11	11
Haplogroup I/M170	northern italian	Italy	17	13-16	13	13	16	25	11	11	13	11	11
Haplogroup I/M170	sardinian	Italy	15	12-15	15	12	16	23	10	11	13	11	11
Haplogroup I/M170	Sardinian	Italy	17	11-13	13	13	15	24	10	11	13	11	11
Haplogroup I/M170	Sicilian	Italy	16	14-15	13	12	19	24	11	11	13	11	13
Haplogroup I/M170	Sicilian	Italy	15	15-15	13	14	18	23	10	12	14	11	11
Haplogroup I/M170	Sicilian	Italy	15	16-17	13	13	17	23	10	12	14	11	11
Haplogroup I/M170	Sicilian	Italy	16	15-15	13	14	17	23	10	12	15	11	11

Haplogroup I/M170	Sicilian Catholics	Italy	16	15-15	13	14	17	23	10	12	15	11	11
Haplogroup I/M170	Sicily	Italy	16	14-16	13	13	17	24	11	11	13	11	13
Haplogroup I/M170	SICILIAN	Italy	15	15-16	13	13	15	23	10	12	15	11	11
Haplogroup I/M170	Venet	Italy	17	13-15	15	14	16	23	10	11	14	11	11
Haplogroup I/M170	Italian	Italy	15	15-15	13	14	18	23	10	13	14	11	11
Haplogroup I/M170	Italian	Italy	14	13-14	14	12	16	22	10	11	13	11	11
Haplogroup I/M170	Italian	Italy	14	13-14	14	12	16	22	10	11	13	11	11
Haplogroup I/M170	Italian	Italy	14	14-14	14	12	16	23	10	11	13	11	11
Haplogroup I/M170	English protestant	United Kingdom	15	14-15	13	13	18	23	10	12	14	11	12
Haplogroup I/M170	england	United Kingdom	14	14-14	14	12	16	21	10	11	13	11	13
Haplogroup I/M170	english	United Kingdom	15	11-16	13	14	17	26	11	11	13	11	11
Haplogroup I/M170	english	United Kingdom	12	13-14	14	12	15	23	10	11	13	11	11
Haplogroup I/M170	english	United Kingdom	14	13-13	15	13	17	22	10	11	14	11	11
Haplogroup I/M170	english	United Kingdom	14	15-15	13	13	18	23	10	12	15	11	11
Haplogroup I/M170	english	United Kingdom	16	15-15	13	13	17	23	10	12	14	11	11
Haplogroup I/M170	english	United Kingdom	16	13-15	14	12	16	23	10	11	13	11	12
Haplogroup I/M170	english	United Kingdom	15	15-15	13	13	18	23	10	12	14	11	14
Haplogroup I/M170	english	United Kingdom	15	15-15	13	14	18	23	10	12	14	11	11
Haplogroup I/M170	english	United Kingdom	14	14-14	14	12	16	22	10	11	13	11	11
Haplogroup I/M170	english	United Kingdom	14	14-15	14	12	16	23	10	11	13	11	11
Haplogroup I/M170	english	United Kingdom	15	13-16	14	12	16	22	11	11	13	11	11
Haplogroup I/M170	english catholic	United Kingdom	14	12-14	14	12	16	22	10	11	13	11	12
Haplogroup I/M170	England	United Kingdom	14	14-14	16	12	16	22	10	11	13	11	11
Haplogroup I/M170	English	United Kingdom	17	13-16	13	13	16	26	11	11	13	11	11
Haplogroup I/M170	English	United Kingdom	16	13-17	13	13	17	22	10	12	13	11	11
Haplogroup I/M170	English	United Kingdom	16	12-15	16	14	16	22	10	11	13	11	12
Haplogroup I/M170	English	United Kingdom	15	15-17	13	14	17	23	10	12	15	11	11
Haplogroup I/M170	English	United Kingdom	16	16-17	13	13	18	22	10	12	14	11	11
Haplogroup I/M170	English	United Kingdom	14	13-14	14	12	17	22	10	11	14	11	11
Haplogroup I/M170	English	United Kingdom	14	14-15	14	12	16	23	10	11	13	11	12
Haplogroup I/M170	English	United Kingdom	14	14-14	14	12	16	22	10	11	13	11	11
Haplogroup I/M170	English	United Kingdom	16	14-15	13	13	16	22	10	12	14	11	11
Haplogroup I/M170	English	United Kingdom	14	14-14	14	12	16	23	10	11	13	11	11
Haplogroup I/M170	English	United Kingdom	15	13-14	16	12	15	22	10	11	14	11	11
Haplogroup I/M170	English	United Kingdom	15	13-14	14	12	16	22	10	11	13	11	11
Haplogroup I/M170	English	United Kingdom	14	13-14	14	12	16	22	10	11	13	11	11
Haplogroup I/M170	English	United Kingdom	14	13-14	14	12	17	23	10	11	13	11	11
Haplogroup I/M170	English	United Kingdom	15	16-16	13	14	16	24	11	12	15	11	11

Haplogroup I/M170	English	United Kingdom	15	15-16	13	14	17	23	10	12	14	11	12
Haplogroup I/M170	English	United Kingdom	15	15-16	13	14	18	23	10	12	14	11	11
Haplogroup I/M170	English	United Kingdom	15	12-13	14	12	16	22	10	11	13	11	11
Haplogroup I/M170	English	United Kingdom	15	13-14	14	11	16	22	10	11	13	11	12
Haplogroup I/M170	English	United Kingdom	14	13-14	16	12	16	22	10	11	13	11	11
Haplogroup I/M170	English	United Kingdom	16	13-17	13	12	17	25	11	11	13	11	11
Haplogroup I/M170	English	United Kingdom	17	15-15	13	13	15	23	10	12	14	11	11
Haplogroup I/M170	English	United Kingdom	14	15-16	14	12	16	23	10	11	13	11	11
Haplogroup I/M170	English	United Kingdom	14	14-15	14	12	16	23	10	11	13	11	12
Haplogroup I/M170	English	United Kingdom	14	12-14	14	12	16	22	10	11	13	11	11
Haplogroup I/M170	English	United Kingdom	14	14-14	14	12	15	23	10	11	13	11	11
Haplogroup I/M170	English	United Kingdom	15	15-16	12	14	17	23	10	12	14	11	11
Haplogroup I/M170	English	United Kingdom	14	13-14	16	12	16	22	10	11	13	11	11
Haplogroup I/M170	English	United Kingdom	15	15-15	13	14	17	23	10	12	15	11	12
Haplogroup I/M170	English	United Kingdom	14	13-13	14	12	16	22	10	11	13	11	11
Haplogroup I/M170	English	United Kingdom	17	12-12	13	14	15	25	9	11	13	11	12
Haplogroup I/M170	English	United Kingdom	16	15-15	13	13	16	23	11	12	14	11	11
Haplogroup I/M170	English	United Kingdom	17	13-15	13	13	17	23	10	13	14	11	11
Haplogroup I/M170	English	United Kingdom	15	13-14	14	12	16	22	10	11	13	11	10
Haplogroup I/M170	English	United Kingdom	14	14-14	14	12	16	23	10	11	13	11	11
Haplogroup I/M170	English	United Kingdom	14	13-14	14	12	16	22	10	11	13	10	11
Haplogroup I/M170	English	United Kingdom	15	15-16	13	13	17	23	10	12	14	11	11
Haplogroup I/M170	English	United Kingdom	14	14-14	14	12	16	23	10	11	13	11	12
Haplogroup I/M170	English	United Kingdom	15	12-15	15	14	16	23	10	11	13	11	12
Haplogroup I/M170	English	United Kingdom	15	14-14	13	14	19	23	10	12	14	11	11
Haplogroup I/M170	English	United Kingdom	15	12-15	14	12	17	22	10	11	13	11	11
Haplogroup I/M170	English	United Kingdom	14	14-14	14	12	16	22	10	11	13	11	11
Haplogroup I/M170	English	United Kingdom	16	16-16	13	12	16	26	11	11	12	11	10
Haplogroup I/M170	English	United Kingdom	14	13-14	14	12	16	23	10	11	13	11	11
Haplogroup I/M170	English	United Kingdom	15	15-16	13	14	18	23	10	12	15	11	13
Haplogroup I/M170	English	United Kingdom	15	16-16	13	13	16	24	10	12	15	11	12
Haplogroup I/M170	English	United Kingdom	14	13-14	14	12	16	23	10	11	13	11	11
Haplogroup I/M170	English	United Kingdom	16	15-15	13	13	16	23	11	12	14	11	13
Haplogroup I/M170	English	United Kingdom	14	13-14	14	12	17	22	10	11	13	11	11
Haplogroup I/M170	English	United Kingdom	14	14-14	14	12	16	24	11	11	13	11	11
Haplogroup I/M170	English	United Kingdom	15	15-15	13	14	17	23	10	12	15	11	11
Haplogroup I/M170	English	United Kingdom	14	13-14	14	12	15	22	10	11	13	11	11
Haplogroup I/M170	English	United Kingdom	13	13-14	14	12	16	22	10	11	14	11	11

Haplogroup I/M170	English	United Kingdom	15	15-16	13	14	17	22	10	12	15	11	11
Haplogroup I/M170	English	United Kingdom	15	14-16	12	14	17	22	10	12	13	11	11
Haplogroup I/M170	English	United Kingdom	15	14-14	13	13	18	23	10	12	13	11	11
Haplogroup I/M170	English	United Kingdom	15	15-16	13	13	18	23	10	12	15	11	12
Haplogroup I/M170	English	United Kingdom	14	13-14	14	12	16	23	10	11	13	11	12
Haplogroup I/M170	English	United Kingdom	15	15-15	13	14	18	23	10	12	14	11	11
Haplogroup I/M170	English	United Kingdom	14	14-14	14	12	16	22	10	11	13	11	11
Haplogroup I/M170	English	United Kingdom	15	13-15	14	12	16	22	10	11	13	11	11
Haplogroup I/M170	English	United Kingdom	14	14-15	14	12	17	23	10	11	13	11	11
Haplogroup I/M170	English	United Kingdom	16	15-15	13	13	16	23	10	12	14	11	11
Haplogroup I/M170	English	United Kingdom	14	14-15	14	12	16	23	10	11	13	11	11
Haplogroup I/M170	English caucasian	United Kingdom	15	15-15	13	14	17	23	10	12	15	11	11
Haplogroup I/M170	English cockney	United Kingdom	17	12-16	13	12	16	26	11	11	14	11	12
Haplogroup I/M170	English Christian	United Kingdom	14	13-14	14	13	16	22	10	11	13	11	12
Haplogroup I/M170	English Protestant	United Kingdom	15	13-15	14	13	16	22	10	11	13	11	11
Haplogroup I/M170	English Protestant	United Kingdom	14	14-14	14	12	18	23	10	11	13	11	12
Haplogroup I/M170	English C of E	United Kingdom	14	13-15	14	12	16	22	10	11	15	11	12
Haplogroup I/M170	English Caucasian	United Kingdom	14	13-14	16	12	16	22	10	11	13	11	11
Haplogroup I/M170	ENGLISH	United Kingdom	16	14-14	14	12	18	23	10	12	15	11	10
Haplogroup I/M170	ENGLISH	United Kingdom	14	14-15	14	12	16	23	10	11	13	11	12
Haplogroup I/M170	ENGLISH	United Kingdom	14	13-14	14	12	16	22	10	11	13	11	11
Haplogroup I/M170	ENGLISH C/E	United Kingdom	15	14-14	14	13	16	22	10	11	13	11	12
Haplogroup I/M170	english	United Kingdom	15	14-15	14	12	17	23	10	11	13	11	11
Haplogroup I/M170	english	United Kingdom	16	13-14	14	12	17	22	10	11	13	11	11
Haplogroup I/M170	english	United Kingdom	15	15-15	13	14	17	23	10	12	14	11	11
Haplogroup I/M170	english	United Kingdom	14	13-15	14	12	16	22	10	11	13	11	11
Haplogroup I/M170	english	United Kingdom	14	13-15	15	12	16	22	10	11	13	11	11
Haplogroup I/M170	english christian	United Kingdom	14	13-14	14	12	16	22	10	11	13	11	12
Haplogroup I/M170	English	United Kingdom	15	13-14	14	13	16	22	10	11	13	11	11
Haplogroup I/M170	English	United Kingdom	14	13-14	14	12	16	22	10	11	13	11	11
Haplogroup I/M170	English	United Kingdom	14	13-14	14	12	16	22	10	11	13	11	11
Haplogroup I/M170	English	United Kingdom	15	12-15	15	14	16	23	10	11	13	11	11
Haplogroup I/M170	English	United Kingdom	14	13-15	14	12	16	23	10	11	13	11	11
Haplogroup I/M170	English	United Kingdom	14	14-15	14	12	16	23	10	11	13	11	11
Haplogroup I/M170	English	United Kingdom	15	13-14	14	12	16	22	10	11	13	11	11
Haplogroup I/M170	English	United Kingdom	14	13-14	14	12	17	23	10	11	13	11	11
Haplogroup I/M170	English	United Kingdom	14	13-14	14	12	17	22	10	11	13	11	12
Haplogroup I/M170	English	United Kingdom	14	14-14	14	12	16	23	10	11	13	11	12

Haplogroup I/M170	English	United Kingdom	14	13-14	16	12	16	22	10	11	13	11	11
Haplogroup I/M170	English	United Kingdom	14	14-14	14	12	16	23	10	11	13	11	11
Haplogroup I/M170	English	United Kingdom	14	14-15	14	12	16	23	10	11	13	11	11
Haplogroup I/M170	English [White Caucasian]	United Kingdom	14	11-14	14	12	16	23	10	11	13	11	11
Haplogroup I/M170	English caucasian	United Kingdom	16	13-16	13	12	16	25	10	11	13	11	12
Haplogroup I/M170	English Christian	United Kingdom	17	14-16	13	13	16	23	10	12	14	11	11
Haplogroup I/M170	English indigenous	United Kingdom	16	15-16	13	14	16	23	10	12	14	11	11
Haplogroup I/M170	ENGLISH	United Kingdom	14	13-14	14	12	16	22	10	11	13	11	11
Haplogroup I/M170	Yorkshire English Anglican	United Kingdom	14	14-15	14	12	17	22	10	11	13	11	11

Y Chromosome Haplogroup/SNP Marker	Father's Ethnicity	Father's Place of Birth	STR Marker										
			19	385 loci	388	389I	389b	390	391	392	393	426	439
Haplogroup J*(xJ2)/12f2.1	french	France	15	12-18	14	14	18	24	10	11	12	11	11
Haplogroup J*(xJ2)/12f2.1	French	France	14	14-17	16	13	16	24	10	11	12	11	11
Haplogroup J*(xJ2)/12f2.1	French	France	14	13-18	17	12	17	23	10	11	12	11	11
Haplogroup J*(xJ2)/12f2.1	German	Germany	14	14-19	13	13	16	23	10	11	13	11	12
Haplogroup J*(xJ2)/12f2.1	German	Germany	14	16-19	16	13	16	25	10	13	12	11	10
Haplogroup J*(xJ2)/12f2.1	german	Germany	14	13-17	16	13	17	26	11	11	13	11	11
Haplogroup J*(xJ2)/12f2.1	German Jewish	Germany	14	13-18	15	14	17	23	10	11	12	11	11
Haplogroup J*(xJ2)/12f2.1	German	Germany	14	13-15	16	13	17	23	10	11	12	11	12
Haplogroup J1/M267	German	Germany	14	13-13	16	13	17	23	10	11	12	11	12
Haplogroup J*(xJ2)/12f2.1	Italian	Italy	14	13-17	16	13	16	23	10	11	12	11	11
Haplogroup J*(xJ2)/12f2.1	Italian	Italy	14	15-18	13	14	18	23	9	11	12	11	12
Haplogroup J*(xJ2)/12f2.1	italian	Italy	14	13-17	16	13	16	24	10	11	12	11	11
Haplogroup J*(xJ2)/12f2.1	Italian	Italy	14	13-20	16	13	18	23	10	11	12	11	11
Haplogroup J*(xJ2)/12f2.1	Italian	Italy	14	13-18	16	14	17	23	10	12	12	11	11
Haplogroup J*(xJ2)/12f2.1	Italian	Italy	14	14-17	17	13	16	23	10	11	12	11	12
Haplogroup J*(xJ2)/12f2.1	ITALIAN	Italy	14	13-18	16	13	17	22	9	11	12	11	12
Haplogroup J*(xJ2)/12f2.1	Italian	Italy	14	14-19	16	13	17	23	10	11	12	11	13
Haplogroup J*(xJ2)/12f2.1	Italian	Italy	14	13-18	15	14	17	23	10	11	12	11	12
Haplogroup J*(xJ2)/12f2.1	Italian	Italy	14	12-18	13	14	16	23	10	11	12	11	11
Haplogroup J*(xJ2)/12f2.1	Italian	Italy	14	13-19	16	13	17	23	10	11	12	11	11
Haplogroup J*(xJ2)/12f2.1	Italian	Italy	14	13-16	16	13	17	22	10	11	12	11	11
Haplogroup J*(xJ2)/12f2.1	italian	Italy	14	13-17	17	13	18	23	11	11	12	11	10
Haplogroup J*(xJ2)/12f2.1	WHITE ITALIAN	Italy	14	10-19	16	13	16	24	10	11	12	11	12
Haplogroup J*(xJ2)/12f2.1	white	Italy	14	13-18	16	13	17	23	11	11	12	11	11
Haplogroup J*(xJ2)/12f2.1	Italian	Italy	14	13-13	17	14	17	22	10	11	12	11	11
Haplogroup J*(xJ2)/12f2.1	Italian	Italy	14	12-18	16	13	17	23	10	11	12	11	11
Haplogroup J*(xJ2)/12f2.1	Italian	Italy	14	13-15	16	14	17	23	10	11	12	11	12
Haplogroup J*(xJ2)/12f2.1	ITALIAN	Italy	15	10-18	15	13	17	24	10	11	12	11	13
Haplogroup J1/M267	Southern Italian	Italy	14	13-18	16	13	16	24	10	11	12	10	12
Haplogroup J1/M267	Italian	Italy	14	13-19	16	13	17	23	10	11	12	11	11
Haplogroup J1/M267	Italian	Italy	14	12-18	13	13	16	23	10	11	12	11	9
Haplogroup J1/M267	Italian	Italy	14	12-18	16	13	17	23	9	11	12	11	11
Haplogroup J1/M267	Italian Catholic	Italy	15	10-19	15	13	17	24	10	11	12	11	13

Haplogroup J1/M267	Italian Caucasian	Italy	14	13-17	15	14	18	23	10	11	10	11	12
Haplogroup J1/M267	italian	Italy	14	14-17	15	13	16	24	9	11	12	11	11
Haplogroup J1/M267	english	United Kingdom	14	13-16	16	13	16	23	10	11	12	11	12

Y Chromosome Haplogroup/SNP Marker	Father's Ethnicity	Father's Place of Birth	STR Marker										
			19	385 loci	388	389I	389b	390	391	392	393	426	439
Haplogroup R1b/M343	French Caucasian	France	14	11-13	12	10	16	24	11	13	13	12	12
Haplogroup R1b/M343	North of France	France	15	11-14	12	13	16	24	11	13	13	12	12
Haplogroup R1b/M343	FRANCE	France	14	11-14	12	13	16	25	11	13	13	12	12
Haplogroup R1b/M343	french	France	14	11-15	12	13	16	24	11	13	13	12	13
Haplogroup R1b/M343	French	France	14	11-14	12	14	17	23	11	13	13	12	12
Haplogroup R1b/M343	french	France	15	11-14	12	13	16	24	10	13	13	12	11
Haplogroup R1b/M343	FRENCH	France	15	11-14	12	13	15	24	11	13	13	12	12
Haplogroup R1b/M343	French	France	14	11-15	12	13	16	24	11	12	13	12	12
Haplogroup R1b/M343	FRENCH	France	15	10-13	12	13	16	24	9	13	13	12	12
Haplogroup R1b/M343	BRETON FRENCH	France	14	11-14	12	13	15	23	10	13	13	12	11
Haplogroup R1b/M343	French	France	14	11-14	12	13	16	24	10	13	13	12	11
Haplogroup R1b/M343	FRENCH	France	14	11-14	12	13	16	24	10	13	13	12	12
Haplogroup R1b/M343	French	France	14	11-14	12	13	17	24	11	13	13	12	11
Haplogroup R1b/M343	french	France	15	12-13	12	13	17	23	10	13	13	12	12
Haplogroup R1b/M343	French	France	14	11-14	12	13	16	23	11	14	13	12	11
Haplogroup R1b/M343	French	France	14	11-14	12	13	16	24	10	13	13	12	12
Haplogroup R1b/M343	French	France	14	11-15	12	13	17	25	10	13	13	12	12
Haplogroup R1b/M343	Breton	France	14	11-14	12	13	16	24	11	13	13	12	12
Haplogroup R1b/M343	White european French	France	14	11-12	12	13	16	24	10	14	13	12	12
Haplogroup R1b/M343	French (Southern)	France	14	11-14	12	13	16	24	11	13	13	12	12
Haplogroup R1b/M343	BRITTANY	France	14	11-14	12	13	16	24	11	13	13	12	11
Haplogroup R1b/M343	french	France	14	11-15	12	13	16	24	10	13	13	12	13
Haplogroup R1b/M343	french	France	15	11-14	12	13	16	24	11	13	13	12	12
Haplogroup R1b/M343	French Catholic	France	14	11-15	12	13	16	23	11	14	13	12	12
Haplogroup R1b/M343	FRENCH	France	14	11-14	12	13	16	23	11	13	13	12	12
Haplogroup R1b/M343	europe (france)	France	14	11-14	12	13	16	24	11	13	13	12	11
Haplogroup R1b/M343	French Catholic	France	14	11-14	12	13	16	25	11	13	13	12	12
Haplogroup R1b/M343	catholic french	France	14	11-14	12	12	16	24	11	13	12	12	13
Haplogroup R1b/M343	FRENCH	France	14	11-15	12	13	16	24	10	13	13	12	13
Haplogroup R1b/M343	German	Germany	14	11-15	12	13	15	23	11	13	13	12	13
Haplogroup R1b/M343	German	Germany	14	11-14	12	13	16	25	11	13	13	12	12
Haplogroup R1b/M343	German	Germany	13	11-15	12	13	16	24	10	13	13	12	13

Haplogroup R1b/M343	German	Germany	15	11-15	12	13	16	24	11	13	13	12	11
Haplogroup R1b/M343	German	Germany	14	11-14	12	13	16	24	11	13	13	12	12
Haplogroup R1b/M343	German	Germany	15	11-14	12	13	16	24	10	13	13	12	12
Haplogroup R1b/M343	German	Germany	14	11-14	12	13	16	24	11	13	13	12	13
Haplogroup R1b/M343	German	Germany	14	11-14	12	13	16	25	10	13	13	12	11
Haplogroup R1b/M343	German Lutheran	Germany	14	11-14	14	13	16	23	11	13	13	12	13
Haplogroup R1b/M343	German	Germany	14	10-15	12	13	16	24	10	15	13	12	13
Haplogroup R1b/M343	German	Germany	14	11-14	12	13	16	23	11	13	13	12	12
Haplogroup R1b/M343	German	Germany	14	11-12	12	13	18	24	11	13	13	12	12
Haplogroup R1b/M343	German	Germany	14	11-14	10	13	16	24	11	13	13	12	13
Haplogroup R1b/M343	Germany	Germany	14	11-14	12	13	16	23	11	13	13	12	12
Haplogroup R1b/M343	german	Germany	15	11-14	13	13	16	24	11	13	13	12	11
Haplogroup R1b/M343	white/German	Germany	14	12-15	12	13	16	24	11	13	13	12	12
Haplogroup R1b/M343	German	Germany	14	11-15	12	13	16	24	10	13	13	12	12
Haplogroup R1b/M343	Germanic	Germany	14	11-15	12	13	16	23	10	13	13	12	12
Haplogroup R1b/M343	German	Germany	14	11-14	12	13	16	23	12	13	13	12	12
Haplogroup R1b/M343	German	Germany	14	11-15	12	13	16	24	11	13	13	12	12
Haplogroup R1b/M343	German	Germany	14	11-15	12	13	16	25	11	13	13	12	13
Haplogroup R1b/M343	Protestant German	Germany	14	11-14	12	13	16	23	11	14	13	12	12
Haplogroup R1b/M343	German	Germany	14	12-14	14	13	16	23	11	13	13	12	13
Haplogroup R1b/M343	German	Germany	14	11-14	12	13	16	23	11	13	13	12	12
Haplogroup R1b/M343	German	Germany	14	12-15	12	13	15	23	11	13	13	12	12
Haplogroup R1b/M343	German	Germany	14	11-14	12	13	16	23	11	14	12	12	12
Haplogroup R1b/M343	German	Germany	15	12-14	12	13	16	23	10	7	13	12	12
Haplogroup R1b/M343	german	Germany	14	11-11	12	13	17	24	11	13	13	12	12
Haplogroup R1b/M343	German	Germany	16	11-14	12	13	17	24	11	13	13	12	11
Haplogroup R1b/M343	German	Germany	14	11-14	12	13	16	23	10	13	13	12	12
Haplogroup R1b/M343	German	Germany	15	11-16	12	13	16	24	10	13	13	12	12
Haplogroup R1b/M343	Italian	Italy	15	10-16	13	13	16	23	11	13	13	12	12
Haplogroup R1b/M343	italian	Italy	14	11-15	12	13	15	23	11	14	12	12	13
Haplogroup R1b/M343	ITALIAN	Italy	14	11-13	12	13	15	23	12	14	12	12	12
Haplogroup R1b/M343	Italian	Italy	14	11-14	12	13	18	24	10	13	13	12	11
Haplogroup R1b/M343	Italian	Italy	14	11-14	12	12	17	24	10	13	13	12	12
Haplogroup R1b/M343	italian	Italy	14	11-14	12	13	16	24	11	13	13	12	13
Haplogroup R1b/M343	Italian	Italy	14	11-11	12	13	16	23	11	13	13	12	12
Haplogroup R1b/M343	Italian	Italy	14	11-14	12	13	16	24	11	13	13	12	12
Haplogroup R1b/M343	Italian	Italy	14	11-14	12	13	16	24	10	13	13	12	12

Haplogroup R1b/M343	italiano	Italy	14	11-15	12	13	16	24	11	13	13	12	13
Haplogroup R1b/M343	Italians	Italy	14	11-14	12	13	16	26	10	13	13	12	11
Haplogroup R1b/M343	Italian	Italy	14	11-14	12	13	16	24	11	13	13	12	12
Haplogroup R1b/M343	italian	Italy	14	11-16	12	13	17	23	11	13	13	12	12
Haplogroup R1b/M343	Italian	Italy	15	11-14	12	13	15	24	11	14	12	12	13
Haplogroup R1b/M343	Italian - Catolic	Italy	14	11-14	12	14	16	24	11	13	13	12	12
Haplogroup R1b/M343	ITALY	Italy	14	14-15	12	13	17	25	12	13	13	12	12
Haplogroup R1b/M343	Italian	Italy	14	11-14	12	13	16	24	11	13	13	13	12
Haplogroup R1b/M343	Italian	Italy	14	11-14	12	13	16	24	11	13	13	12	13
Haplogroup R1b/M343	Italian	Italy	15	11-13	12	13	17	24	10	13	13	12	11
Haplogroup R1b/M343	Italian	Italy	14	11-13	12	13	16	25	10	14	12	12	12
Haplogroup R1b/M343	Italian	Italy	14	12-14	12	13	16	24	13	13	13	12	11
Haplogroup R1b/M343	ITALIAN	Italy	14	11-14	12	13	16	25	11	13	12	12	12
Haplogroup R1b/M343	Italian	Italy	14	11-15	12	12	16	24	11	13	13	12	12
Haplogroup R1b/M343	Italian	Italy	15	11-14	12	13	16	24	11	13	13	12	12
Haplogroup R1b/M343	italian	Italy	14	11-14	12	13	17	23	11	12	11	12	12
Haplogroup R1b/M343	ITALY	Italy	15	11-15	13	13	16	24	11	13	14	12	13
Haplogroup R1b/M343	Italian	Italy	14	12-15	11	13	16	24	11	13	12	12	12
Haplogroup R1b/M343	English	United Kingdom	14	11-14	12	13	17	23	11	13	13	12	12
Haplogroup R1b/M343	English	United Kingdom	15	11-16	12	13	16	24	10	13	12	12	11
Haplogroup R1b/M343	English	United Kingdom	14	11-14	12	13	16	23	11	13	13	12	12
Haplogroup R1b/M343	English	United Kingdom	14	14-14	12	13	17	24	11	13	13	12	12
Haplogroup R1b/M343	English	United Kingdom	14	11-14	12	13	16	24	10	14	13	12	12
Haplogroup R1b/M343	English	United Kingdom	14	11-14	12	13	16	24	10	13	13	12	12
Haplogroup R1b/M343	English	United Kingdom	14	12-13	12	13	16	24	11	13	13	12	11
Haplogroup R1b/M343	English	United Kingdom	14	11-14	12	15	16	23	11	13	13	12	11
Haplogroup R1b/M343	English	United Kingdom	14	11-13	12	13	15	24	11	13	13	12	11
Haplogroup R1b/M343	English	United Kingdom	14	11-14	12	13	15	23	12	13	13	12	12
Haplogroup R1b/M343	English	United Kingdom	14	11-14	12	13	17	24	11	13	13	12	13
Haplogroup R1b/M343	English	United Kingdom	14	12-15	12	14	16	24	11	13	13	12	11
Haplogroup R1b/M343	English	United Kingdom	14	11-15	12	14	16	24	11	13	13	12	11
Haplogroup R1b/M343	English	United Kingdom	14	11-14	12	13	16	24	11	13	14	12	11
Haplogroup R1b/M343	English	United Kingdom	15	11-14	12	13	17	23	12	13	13	12	12
Haplogroup R1b/M343	English	United Kingdom	14	11-14	12	13	16	23	10	14	13	12	12
Haplogroup R1b/M343	English	United Kingdom	14	11-15	12	13	17	24	10	13	13	12	12
Haplogroup R1b/M343	English	United Kingdom	15	11-14	12	13	15	22	11	13	13	12	11
Haplogroup R1b/M343	English	United Kingdom	14	11-15	11	13	16	23	11	13	13	12	11

Haplogroup R1b/M343	English	United Kingdom	14	11-15	12	13	16	23	10	13	13	12	11
Haplogroup R1b/M343	English	United Kingdom	15	11-14	12	13	16	24	11	13	13	12	11
Haplogroup R1b/M343	English	United Kingdom	14	12-12	12	13	16	24	10	13	13	12	12
Haplogroup R1b/M343	English	United Kingdom	14	11-15	12	13	16	23	11	13	13	12	12
Haplogroup R1b/M343	English	United Kingdom	14	11-14	12	13	15	23	11	13	13	12	11
Haplogroup R1b/M343	English	United Kingdom	14	11-14	12	14	16	23	11	13	13	12	12

Participants provided information about their father’s ethnicity and father’s place of birth; this is reproduced as provided, and thus varies from individual to individual. Data were included from consenting participants from the Arabian Peninsula, France, Germany, Italy and the UK if the participant’s country of residence and father’s birthplace were the same, and (for the European individuals) if they belonged to haplogroup I, J*(xJ2) or R1b.
